# Artificial Intelligence for Precision Antiarrhythmic Drug Therapy in Atrial Fibrillation: From Recurrence Prediction to Comparative Treatment Selection

**DOI:** 10.3390/medicina62091783

**Published:** 2026-09-16

**Authors:** Alina Scridon, Vasile-Bogdan Halațiu, Dan-Alexandru Cozac

**Affiliations:** 1 Department of Physiology, George Emil Palade University of Medicine, Pharmacy, Science, and Technology of Targu Mures, 540142 Târgu Mureș, Romaniadan-alexandru.cozac@umfst.ro (D.-A.C.); 2 Department of Cardiology, Emergency Clinical County Hospital Târgu Mureș, 540136 Târgu Mureș, Romania

**Keywords:** antiarrhythmic drugs, artificial intelligence, atrial fibrillation, digital twin, machine learning, precision medicine

## Abstract

Rhythm control therapy has an important role in atrial fibrillation (AF) management, and antiarrhythmic drugs (AADs) remain essential for pharmacological cardioversion, maintenance of sinus rhythm, reduction in AF burden, and treatment before or after catheter ablation. However, their efficacy varies substantially among patients, while proarrhythmia, organ toxicity, drug interactions, and treatment discontinuation frequently limit their use. Current drug selection therefore relies mainly on safety-based exclusion based on structural heart disease, ventricular function, coronary disease, renal or hepatic function, and baseline conduction and repolarization characteristics, rather than on individualized prediction of comparative therapeutic benefit. This narrative review examines the potential role of artificial intelligence (AI), machine learning (ML), computational electrophysiology, and cardiac digital twins across the AAD treatment pathway. Particular attention is given to patient selection, comparative drug choice, prediction of cardioversion success and sinus-rhythm maintenance, dose optimization, proarrhythmia assessment, extracardiac toxicity, and longitudinal safety surveillance. AI can potentially integrate clinical, electrocardiographic (ECG), imaging, wearable, genomic, and pharmacological data to estimate patient-specific efficacy and toxicity. ML models have already demonstrated the feasibility of predicting drug-induced QT prolongation from electronic health records and detecting ECG signatures associated with drug-induced arrhythmic risk. Moreover, patient-specific AF digital twins have been used to simulate electrophysiological responses to amiodarone and identify patients with different subsequent rhythm outcomes. Nevertheless, most available applications remain retrospective, single-center, non-comparative, or proof-of-concept, and few directly support selection among alternative AADs. Most are prognostic, estimate outcomes under observed care, or predict drug-specific toxicity; models that estimate outcomes under alternative AADs remain the essential missing element. AI-supported antiarrhythmic therapy represents a promising transition from population-based prescribing toward individualized estimation of efficacy, toxicity, and monitoring requirements. Its clinical adoption will require multicenter external validation, causal treatment-effect modeling, prospective workflow evaluation, randomized impact trials, transparent uncertainty reporting, and continued clinician oversight.

## 1. Introduction

Rhythm control has moved from a predominantly symptom-directed option toward an earlier and more integrated component of atrial fibrillation (AF) management. The Early Treatment of Atrial Fibrillation for Stroke Prevention Trial (EAST-AFNET 4) demonstrated that early rhythm control therapy, delivered through antiarrhythmic drugs (AADs), cardioversion, and catheter ablation, according to clinical needs, reduced major cardiovascular outcomes compared with usual care in patients with recently diagnosed AF and cardiovascular conditions [1]. Guidelines accordingly recommend timely consideration of rhythm control within a pathway that also addresses thromboembolic risk, comorbidities, and repeated reassessment of symptoms and AF burden [2,3]. Catheter ablation has become an increasingly important first-line option, particularly in selected patients with symptomatic paroxysmal AF, with lower recurrence after initial cryoballoon ablation than with AAD therapy [4,5]. Radiofrequency and, more recently, pulsed-field ablation are alternative energy sources, but comparative selection among ablation modalities is outside the scope of this review, which focuses on pharmacological therapy [2,3]. Nevertheless, AADs remain indispensable for pharmacological cardioversion, maintenance of sinus rhythm, treatment before or after ablation, and patients who prefer, cannot undergo, or have limited access to invasive therapy [2,6].

Selection among AADs, however, remains only modestly personalized. Current algorithms identify which drugs can be used safely rather than which drug is most likely to benefit a particular patient. Prescribing is therefore organized around the presence or absence of structural heart disease, left ventricular systolic function, coronary artery disease (CAD), renal and hepatic function, baseline bradycardia or conduction disease, QT interval, and established contraindications, interactions, and organ-specific toxicities [2,3,6]. These safeguards are essential, but they divide patients into broad eligibility categories and do not capture interindividual differences in atrial substrate, AF mechanism and burden, autonomic influences, pharmacokinetics, adherence, or susceptibility to treatment failure and adverse effects. Consequently, patients who appear similar within guideline algorithms may experience markedly different rhythm outcomes, tolerability, and monitoring requirements.

**Figure 1:**
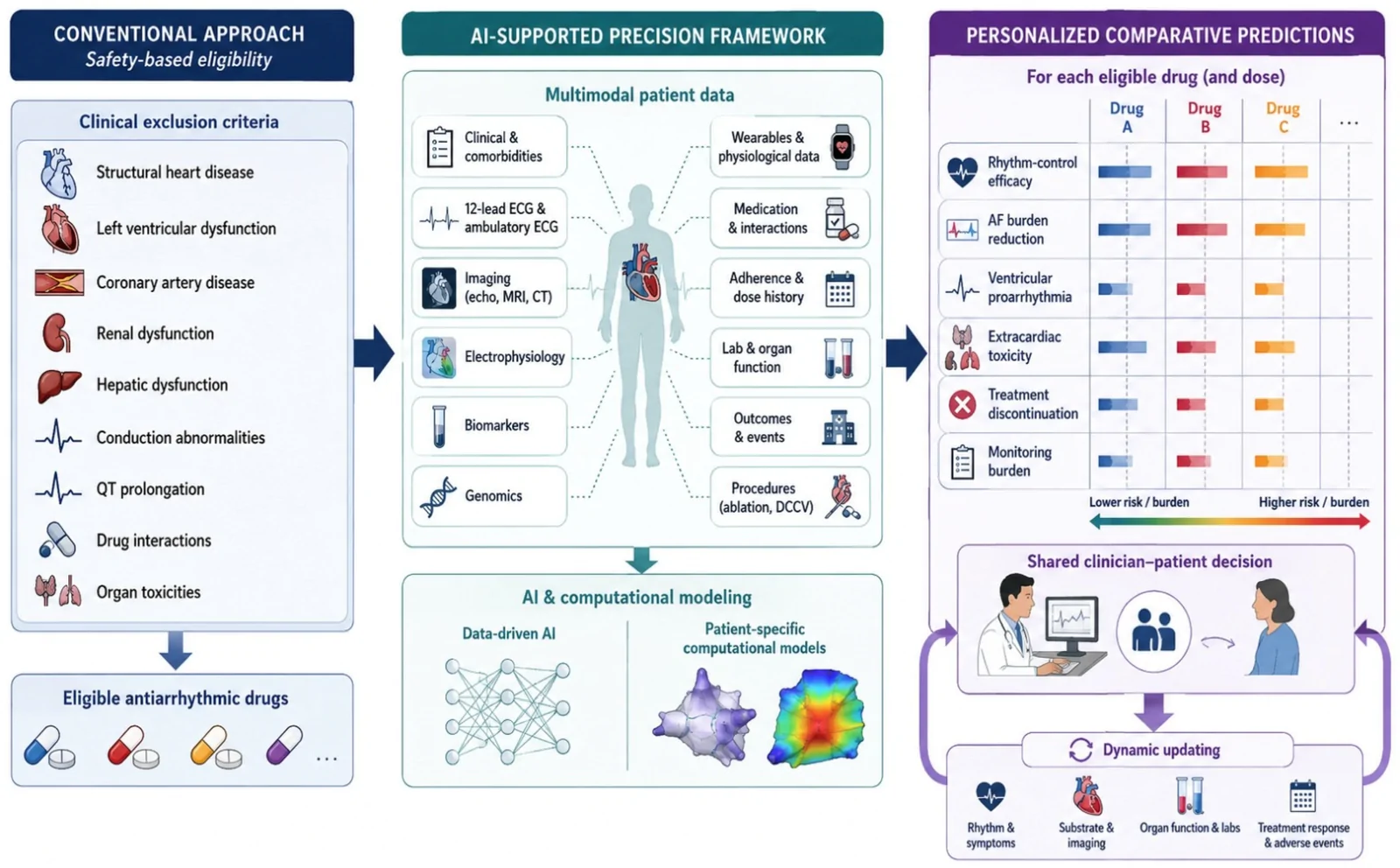
From safety-based antiarrhythmic drug (AAD) eligibility to artificial intelligence (AI)-supported precision pharmacotherapy in atrial fibrillation (AF). Conventional selection identifies drugs that can be used safely according to clinical exclusion criteria and contraindications. The proposed framework adds multimodal patient data and computational modeling to compare the expected benefits, risks, and monitoring needs of the remaining treatment options. These comparative estimates would inform, but not replace, shared clinician-patient decision-making and would be dynamically updated as the patient’s rhythm, substrate, comorbidities, organ function, and treatment response evolve. CT—computed tomography; DCCV—direct current cardioversion; MRI—magnetic resonance imaging.

Artificial intelligence (AI) offers a potential route from safety-based eligibility toward comparative, patient-specific pharmacotherapy. Its principal opportunity is not merely to classify patients as likely responders or non-responders, but also to integrate clinical, ECG, imaging, laboratory, wearable, genomic, and longitudinal treatment data to estimate the individual balance among rhythm control efficacy, AF recurrence or burden, ventricular proarrhythmia, extracardiac toxicity, drug interactions, and treatment burden. Mechanistic computational models may complement data-driven AI by simulating how patient-specific anatomy and electrophysiology respond to alternative drugs and doses. Early proof-of-concept work has used AF digital twins to simulate amiodarone response, although the evidence remains retrospective and far from routine clinical implementation [7]. Figure 1 contrasts conventional exclusion-based selection with an AI-supported framework that produces drug- and dose-specific estimates updated as the patient’s substrate and organ function evolve.

Throughout this review, we distinguish three types of AI models. Prognostic models estimate the risk of an outcome under the care a patient actually receives. Drug-specific safety models estimate the risk of toxicity after exposure to a particular drug. Comparative causal models estimate how the same patient would be expected to fare with different eligible treatments. Only this third model type could guide individualized drug selection. Validated evidence for this use is currently lacking. This narrative review examines how AI, machine learning (ML), computational electrophysiology, and cardiac digital twins could support decisions across the AF antiarrhythmic treatment pathway, from patient and drug selection to dose optimization, efficacy prediction, toxicity surveillance, and adaptive reassessment. Detailed applications of AI to AF screening, diagnosis, stroke prediction, or ablation planning are outside its scope, except when they directly inform pharmacological rhythm control. This article emphasizes distinguishing demonstrated capabilities from conceptual or preclinical applications and defining the evidence required before AI-supported AAD prescribing can enter clinical practice.

## 2. Review Methodology

This review was designed as a focused, state-of-the-art narrative synthesis addressing the use of AI and computational modeling to support AAD therapy in AF. Reporting was informed by the principles of the Scale for the Assessment of Narrative Review Articles (SANRA) [8,9], which is a quality-appraisal instrument for narrative review and does not itself prescribe a search or selection protocol. Relevant literature was identified through complementary searches of PubMed/MEDLINE, Scopus, and Web of Science from database inception through 31 July 2026. Institute of Electrical and Electronics Engineers (IEEE) Xplore was additionally searched for engineering and computational studies that may not be comprehensively indexed in biomedical databases. Searches were supplemented by backward reference screening of eligible articles and relevant reviews, forward citation tracking, and targeted searches for major clinical guidelines, consensus statements, regulatory documents, and pivotal studies. A complete database-specific search strategy is provided in Supplementary Table S1.

Search concepts combined controlled vocabulary, where available, with free-text terms related to “atrial fibrillation,” “antiarrhythmic drug,” “rhythm control,” “artificial intelligence,” “machine learning,” “deep learning,” “treatment response,” “precision pharmacotherapy,” “pharmacovigilance,” “proarrhythmia,” “drug toxicity,” “computational electrophysiology,” “in silico trial,” “virtual patient,” and “digital twin.” Drug-specific terms, including flecainide, propafenone, sotalol, dofetilide, dronedarone, and amiodarone, were incorporated where appropriate.

Evidence was considered eligible if it addressed AI, ML, or computational electrophysiology, or digital-twin methods in relation to AAD, rhythm control, proarrhythmia, or drug-related toxicity in AF, or if it provided the clinical, pharmacological, methodological, or regulatory context required to interpret those studies. Records addressing AF screening, diagnosis, stroke risk prediction, or catheter ablation planning without a pharmacological component were not retained, consistent with the stated scope of this review. Because of substantial heterogeneity in populations, data modalities, drugs, outcomes, and modeling approaches, no quantitative pooling was undertaken. Findings were synthesized thematically and interpreted according to study design, validation level, clinical proximity, and important potential sources, with clear separation of established evidence, proof-of-concept findings, and proposed future applications.

## 3. Why Antiarrhythmic Drug Therapy Needs Greater Precision

### 3.1. Heterogeneous Efficacy of Antiarrhythmic Drugs

The response to AAD therapy varies substantially across drugs, patients, clinical settings, and therapeutic objectives. For acute pharmacological cardioversion, efficacy depends on the agent selected, AF duration, underlying cardiac disease, atrial remodeling, route and timing of administration, and the definition and observation window used for successful conversion [2,6]. A drug that rapidly terminates recent-onset AF may be less effective in persistent AF or unsuitable in the presence of structural heart disease. Conversely, a drug selected for long-term maintenance may not be the optimal agent for rapid conversion.

Heterogeneity persists after restoration of sinus rhythm. AADs reduce AF recurrence compared with placebo or no treatment, but no agent reliably prevents recurrence in all patients, and comparative efficacy differs meaningfully among drugs [10]. Amiodarone, for example, has generally been more effective than sotalol, propafenone, or dronedarone for maintaining sinus rhythm, but its greater efficacy is offset by cumulative extracardiac toxicity and frequent treatment discontinuation [11,12]. Therapeutic benefit also extends beyond binary freedom from recurrence. Some patients experience fewer or shorter episodes, lower AF burden, slower ventricular rates during recurrence, improved symptoms, or reduced need for cardioversion despite continued intermittent AF. Others remain symptomatic despite apparently modest rhythm burden, illustrating that rhythm outcomes and patient-perceived benefit are related but not interchangeable.

The effectiveness of AAD therapy also varies according to treatment context. Drugs may facilitate electrical cardioversion, prevent immediate recurrence after cardioversion, or suppress arrhythmias during periods of heightened vulnerability. After catheter ablation, short-term AAD therapy reduces early atrial arrhythmias in some populations but has generally shown limited ability to prevent late recurrence once treatment is withdrawn [13,14]. In contrast, continued therapy beyond the blanking period may benefit selected patients after pulmonary vein isolation, particularly when the previously ineffective drug becomes effective after modification of the arrhythmogenic substrate [15]. These apparently discordant findings indicate that post-ablation drug efficacy depends on AF phenotype, ablation result, timing, treatment duration, and patient selection rather than on a uniform class effect.

### 3.2. The Safety-Efficacy Trade-Off

Current AAD selection is necessarily dominated by safety. Structural heart disease, ventricular dysfunction, ischemic heart disease, conduction abnormalities, QT prolongation, renal or liver impairment, electrolyte disturbances, and interacting medications exclude or constrain several therapeutic options [2,3,6]. This approach prevents avoidable harm, but the safest eligible drug is not necessarily the most effective drug for an individual patient. A relatively well-tolerated agent may provide inadequate rhythm control, whereas a more effective drug may impose greater risks of bradyarrhythmia, ventricular proarrhythmia, organ toxicity, or intensive monitoring. The comparison between amiodarone and dronedarone illustrates this tension: amiodarone provides greater rhythm efficacy, while dronedarone generally has a more favorable non-thyroid and non-pulmonary toxicity profile in appropriately selected patients [6]. The clinically relevant objective is therefore not maximal efficacy or minimal toxicity in isolation, but the best individualized balance among efficacy, safety, treatment burden, and patient preference.

### 3.3. Therapeutic Failure as a Multidimensional Outcome

Labeling AAD therapy simply as “successful” or “unsuccessful” obscures several biologically and clinically distinct outcomes. Primary pharmacological non-response may reflect an inappropriate drug-mechanism match, advanced atrial remodeling, inadequate exposure, or inability to achieve a therapeutic dose. Partial response may include reduced AF burden or symptoms without complete arrhythmia suppression and may still represent meaningful benefit. Early recurrence after cardioversion can indicate incomplete suppression of a vulnerable atrial substrate, whereas late recurrence may result from progressive fibrosis, chamber dilation, accumulating comorbidity, or changes in autonomic and metabolic modifiers.

**Figure 2:**
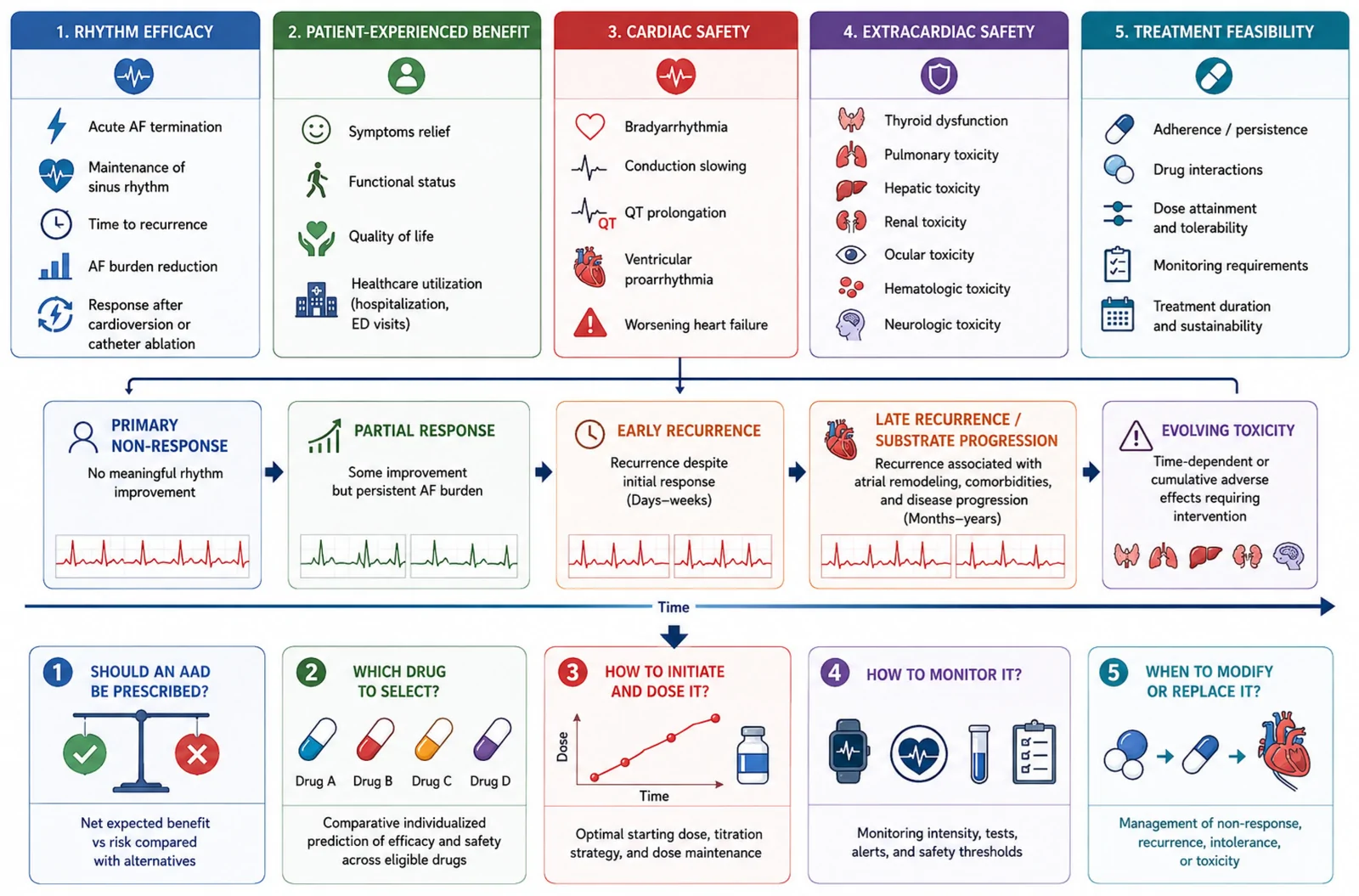
Antiarrhythmic drug (AAD) response as a multidimensional and time-dependent therapeutic outcome. The figure replaces the conventional binary classification of AAD therapy as “success” or “failure” with five interdependent domains: rhythm efficacy, patient-experienced benefit, cardiac safety, extracardiac safety, and treatment feasibility. Rhythm efficacy encompasses acute atrial fibrillation (AF) termination, maintenance of sinus rhythm, time to recurrence, AF burden, and response after cardioversion or catheter ablation. Patient-experienced benefit includes symptoms, functional status, quality of life, and healthcare utilization. Safety incorporates bradyarrhythmia, conduction slowing, QT prolongation, ventricular proarrhythmia, and organ-specific toxicity. Treatment feasibility includes adherence, drug interactions, dose attainment, monitoring requirements, and treatment duration. The longitudinal axis distinguishes primary non-response, partial response, early recurrence, late recurrence associated with substrate progression, and evolving toxicity. The figure culminates in the five clinical decisions that an artificial intelligence (AI)-supported system must address: whether to prescribe an AAD, which drug to select, how to initiate and dose it, how to monitor it, and when to modify or replace it. ED—Emergency Department.

Treatment may also fail because adverse effects prevent adequate dosing; adherence may decline because of complex schedules, monitoring requirements, or perceived toxicity; renal or hepatic function may change drug exposure; or interacting therapies may alter pharmacokinetics and electrophysiological risk. Some apparent failures arise from inappropriate treatment duration; for example, discontinuing an effective drug too early, or continuing an ineffective or toxic drug after the therapeutic objective has changed. Consequently, efficacy, tolerability, adherence, safety, and temporal disease evolution should be assessed jointly rather than collapsed into a composite, with discontinuation, ablation, and death treated as competing events rather than as censoring. This multidimensional and time-dependent concept of AAD response is summarized in Figure 2.

### 3.4. Limitations of Conventional Clinical Algorithms and Expected Role of Artificial Intelligence

Guidelines and consensus documents appropriately categorize patients into broad groups defined by ventricular function, structural heart disease, CAD, and major contraindications [2,3]. These algorithms identify acceptable options but generally do not estimate the comparative probability that flecainide, propafenone, dronedarone, sotalol, dofetilide, or amiodarone will achieve a particular patient’s therapeutic goals. Nor do they quantify how to weigh expected efficacy against risks of proarrhythmia, extracardiac toxicity, discontinuation, and monitoring burden. Treatment selection therefore remains dependent on population-average evidence, clinician experience, local availability, and sequential therapeutic trial and error.

We propose that a clinically useful AI system should move beyond recurrence prediction and support five linked decisions. However, this framework is put forward here for discussion rather than derived from any existing consensus statement:

1. Should this patient receive an antiarrhythmic drug?
2. Which eligible drug offers the most favorable individualized benefit-risk balance?
3. What dose and initiation strategy should be used?
4. How should efficacy, adherence, and cardiac and extracardiac toxicity be monitored?
5. When should treatment be modified, discontinued, or replaced by catheter ablation?

Answering these questions requires comparative, longitudinal, and uncertainty-aware models capable of recognizing that both the patient and the AF substrate evolve over time.

## 4. Determinants of Individual Antiarrhythmic Drug Response

**Figure 3:**
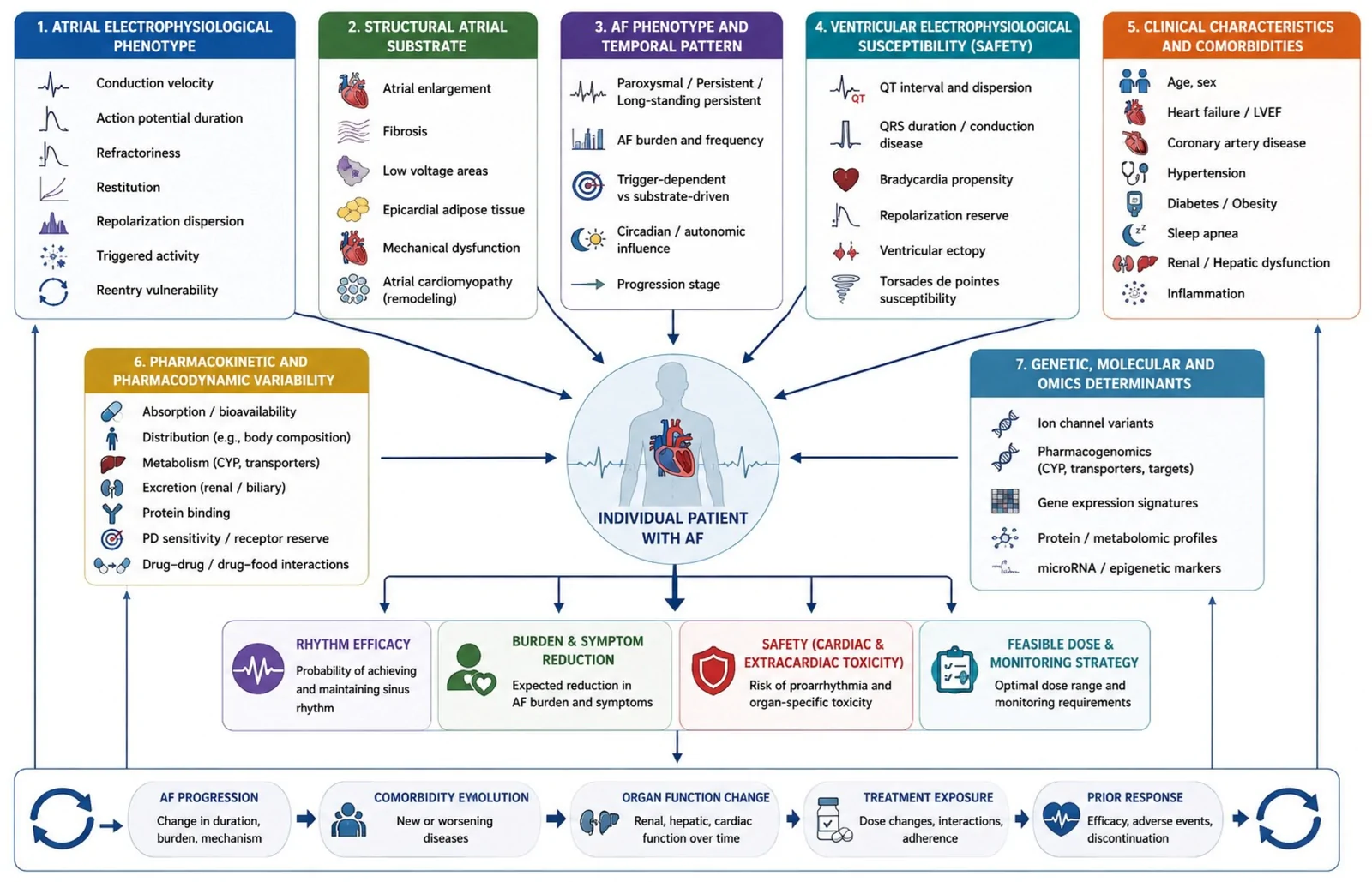
Multilevel determinants of individual antiarrhythmic drug (AAD) response in atrial fibrillation (AF). Seven biological and clinical domains converge on four individualized therapeutic outputs: probability of rhythm efficacy, expected reduction in AF burden and symptoms, cardiac and extracardiac toxicity, and feasible dose and monitoring strategy. Bidirectional arrows indicate that AF progression, comorbidity evolution, organ function, treatment exposure, and prior response modify these determinants over time. CYP—cytochrome; LVEF—left ventricular ejection fraction; PD—pharmacodynamic; RNA—ribonucleic acid.

Individual response to an AAD emerges from the interaction between the drug’s electrophysiological and pharmacological actions and a patient-specific, time-varying biological substrate. The same diagnosis of AF may therefore encompass markedly different mechanisms of initiation and maintenance, structural remodeling, ventricular safety reserves, drug exposure, and molecular determinants. These variables provide the biological features future AI models must represent to predict treatment response rather than reproduce conventional eligibility rules. The interacting determinants of individual AAD response are summarized in Figure 3.

### 4.1. Atrial Electrophysiological Phenotype

The atrial electrophysiological phenotype determines whether a drug meaningfully disrupts the mechanisms sustaining AF. Conduction velocity and effective refractory period jointly influence excitation wavelength; sodium-channel blockade predominantly slows conduction, whereas potassium-channel blockade generally prolongs action-potential duration and refractoriness. Their net antiarrhythmic or proarrhythmic effect consequently depends on baseline conduction, rate dependence, tissue excitability, and spatial heterogeneity. Chronic AF alters calcium and potassium currents producing a substrate that may respond differently from non-remodeled atria [16,17]. Steep restitution, repolarization dispersion, conduction slowing at short cycle lengths, and regional discontinuities can promote wavebreak and stable reentry. Human mapping studies have linked low voltage remodeling and rate-dependent conduction slowing to reentrant drivers sustaining AF [18]. Conversely, trigger-dominant AF may depend more strongly on pulmonary vein automaticity, delayed afterdepolarizations, or abnormal calcium release. Thus, a drug effective against triggered activity may perform poorly when fibrosis-supported reentry predominates, while use-dependent sodium channel blockade may be effective, or hazardous, according to activation rate and conduction reserve.

### 4.2. Structural Atrial Substrate

Atrial enlargement increases the available path length for reentry and commonly accompanies fibrosis, anisotropic conduction, and mechanical dysfunction. Fibrosis separates myocyte bundles, creates conduction discontinuities, and increases spatial dispersion, reducing the likelihood that modifying a single ion current will fully suppress AF [19]. Delayed-enhancement cardiac magnetic resonance has shown a graded association between left atrial fibrosis and arrhythmia recurrence after ablation, independent of conventional clinical variables [20]. Electroanatomical low voltage areas, although influenced by rhythm, electrode characteristics, wavefront direction, and wall thickness, similarly identify advanced electrical degeneration and slow conduction [18]. Epicardial adipose tissue may add a local inflammatory, fibrotic, and infiltrative influence. Moreover, greater periatrial adiposity has been associated with adjacent conduction disturbance and adverse rhythm outcomes after pulmonary vein isolation in patients with AF [21]. Atrial dimensions, strain, reservoir and booster pump function, fibrosis, and low voltage extent should therefore be treated as complementary manifestations of atrial cardiomyopathy rather than interchangeable markers [22]. Collectively, they may identify a substrate in which AADs reduce burden without achieving durable elimination of AF.

### 4.3. Atrial Fibrillation Phenotype and Temporal Pattern

Clinical AF classification is an imperfect but useful proxy for mechanism and disease stage. Recent-onset and paroxysmal AF are more often trigger-dependent, whereas long-standing persistent AF more commonly reflects widespread electrical and structural remodeling. AF duration before cardioversion, cumulative burden, episode length, and time since diagnosis may therefore modify both conversion success and maintenance efficacy. Experimental evidence that sustained AF progressively shortens refractoriness established the principle that “AF begets AF” [16]. Temporal context also matters since early recurrence after cardioversion may reflect transient electrical instability or immediate trigger reactivation, whereas late recurrence may indicate continuing substrate progression. After ablation, pulmonary vein isolation changes the mechanism that an AAD must control, and a previously ineffective drug may become useful when trigger input has been reduced. Autonomically mediated, exercise-related, nocturnal, inflammatory, postoperative, and sleep-related patterns may likewise indicate distinct combinations of triggers and substrate, with autonomic imbalance contributing to atrial ectopic activity and the temporal clustering of AF episodes [23]. Untreated obstructive sleep apnea, for example, has been associated with substantially more AF recurrence after cardioversion [24].

### 4.4. Ventricular Electrophysiological Susceptibility

Atrial efficacy cannot be separated from ventricular safety. Baseline QT interval, QRS duration, bundle branch or atrioventricular conduction disease, bradycardia, ventricular ectopy, and impaired repolarization reserve determine how strongly an AAD can be dosed before risk becomes unacceptable. Sodium channel blockers may produce concentration- and rate-dependent QRS widening, particularly when conduction reserve is limited. Rapid delayed rectifier potassium current blocking agents may cause excessive QT prolongation and *torsades de pointes* when repolarization reserve is reduced by female sex, heart failure, bradycardia, hypokalemia, renal dysfunction, genetic susceptibility, or concomitant QT-prolonging drugs [25,26]. QT duration alone incompletely reflects risk; beat-to-beat repolarization instability and interactions among multiple latent vulnerabilities may be more informative. Precision selection must therefore estimate atrial benefit and ventricular hazard simultaneously.

### 4.5. Clinical and Comorbidity-Related Determinants

Age, sex, and comorbidity influence atrial remodeling, electrophysiological reserve, and drug exposure simultaneously. Heart failure, ischemic heart disease, and valvular disease alter both the AF substrate and the safety of specific drug classes, while obesity, sleep apnea, diabetes, systemic inflammation, thyroid disease, and other metabolic conditions promote substrate progression and can undermine rhythm control durability. Organ function acts principally through exposure: flecainide, sotalol, and dofetilide are particularly sensitive to renal function, whereas propafenone, dronedarone, and amiodarone are strongly affected by hepatic metabolism and clinically important drug interactions [6]. Body composition, transporter activity, and polypharmacy can alter concentrations without changing the prescribed dose, and pharmacodynamic effects are also rate-dependent: flecainide-related conduction slowing increases at faster heart rates, whereas reverse-use-dependent repolarization prolongation with some class III agents is most pronounced during bradycardia. Electrolyte disturbances, acute illness, and changing volume status can transform a previously acceptable regimen into a proarrhythmic one. These variables are dynamic rather than baseline-only covariates and should be updated during therapy [6].

### 4.6. Genetic and Molecular Determinants

Genetic variation may influence both atrial mechanism and drug disposition. In two AF registry cohorts, the chromosome 4q25 variant rs10033464 near *PITX2* independently modified symptomatic response and recurrence during AAD therapy [27]. Experimental work subsequently showed that *PITX2* expression alters atrial resting membrane potential and the effectiveness of sodium channel blockers, providing a mechanistic link between genotype and drug action [28]. In parallel, long-standing arterial hypertension has been associated with atrial *PITX2* downregulation in a model of spontaneous atrial tachyarrhythmias, suggesting that acquired substrate progression may also modify expression of this developmental regulator rather than germline variation alone [29]. Variants in ion channel genes may modify excitability, calcium handling, conduction, or repolarization reserve, whereas cytochrome P450 polymorphisms can alter metabolism. However, evidence supporting routine pharmacogenomic prescribing remains limited and sometimes inconsistent. A recent AF cohort illustrates continuing uncertainty regarding the clinical relevance of CYP2D6 phenotype for flecainide efficacy and toxicity [30]. Circulating proteins, inflammatory markers, metabolites, transcriptomic signatures, and polygenic scores may eventually refine phenotyping, but most remain markers of AF susceptibility or recurrence rather than validated predictors of comparative AAD response. Their greatest value may emerge through multimodal integration with ECG, imaging, drug-exposure, and longitudinal outcome data.

## 5. Data and Artificial Intelligence Approaches for Precision Antiarrhythmic Therapy

Precision antiarrhythmic therapy requires data that capture baseline eligibility, atrial substrate, drug exposure, treatment response, and evolving toxicity. Useful AI systems must therefore combine complementary data streams and match each modeling approach to a defined therapeutic question.

### 5.1. Clinical and Electronic Health Record Data

Electronic health records provide a longitudinal representation of the factors governing AAD use. Diagnoses, ventricular function, medication, prior cardioversions, previous drug responses, and adverse events can be assembled into time-stamped patient histories suited to predicting drug eligibility, dose constraints, interaction risk, treatment discontinuation, and toxicity. ML models applied to harmonized electronic health record data have shown that integrated clinical variables can identify patients susceptible to drug-induced QT prolongation, although transportability across institutions remains uncertain [31]. Such models must also distinguish biological predictors from documentation practices, local formularies, clinician preferences, and confounding by indication.

### 5.2. Electrocardiographic Data

The ECG is the most direct non-invasive record of the electrophysiological effects that AADs are intended to modify. The standard 12-lead recording provides rhythm, atrial activity, ventricular rate, and conduction and repolarization intervals. Ambulatory ECG extends this to episode burden, onset and termination patterns, ectopy, pauses, and rate-dependent QRS or QT changes. High-resolution or signal-averaged recordings may capture lower amplitude atrial activity not represented by conventional measurements.

Deep learning can extract waveform features beyond manually measured intervals. Convolutional neural networks have outperformed QTc alone in identifying exposure to QT-altering drugs and patterns associated with drug-induced *torsades de pointes* [32]. More recent work reconstructed QT/QTc information from single-lead continuous recordings and identified clinically important prolongation after class III AAD initiation, illustrating a transition from occasional ECG snapshots to longitudinal safety surveillance [33]. Serial pre- and post-initiation ECGs may be especially informative because within-patient changes can reveal pharmacodynamic response before clinical toxicity occurs.

### 5.3. Continuous Monitoring and Wearable Data

Wearable and implantable sensors can redefine therapeutic response as a time-varying phenotype. Continuous or near-continuous ECG and photoplethysmography can quantify AF burden, episode duration, ventricular rate patterns, circadian clustering, and changes after drug initiation or dose adjustment. Prospective smartwatch studies have demonstrated feasible algorithm-guided estimation of AF burden, although motion artifact, missingness, skin-device contact, and reduced data quality during activity remain important limitations [34]. In a prospective study by Zhao and collaborators, patients admitted for catheter ablation wore a photoplethysmography-based smartwatch alongside a reference patch ECG; interval-level sensitivity was 96.3% and specificity 99.5%, and estimated AF burden agreed closely with the ECG reference (mean difference −0.59%, 95% limits of agreement −7.9% to +6.7%). The study validated AF burden measurement against a reference standard over a short monitoring period, not its use to guide AAD initiation or dose adjustment [34]. On the other hand, activity, sleep, resting heart rate, heart rate recovery, and cautiously interpreted autonomic surrogates may help identify trigger-related recurrence or treatment intolerance. Sensor patterns may suggest non-adherence but should not establish it without corroboration.

### 5.4. Cardiac Imaging and Electro-Anatomical Data

Echocardiography supplies atrial dimensions, ventricular function, valvular disease, and atrial mechanical indices that influence eligibility and rhythm-control durability. Cardiac computed tomography defines atrial anatomy and epicardial adipose tissue; late gadolinium enhancement magnetic resonance characterizes fibrosis; and electroanatomical maps represent conduction slowing and substrate complexity. A study integrating deep learning segmentation, late gadolinium enhancement magnetic resonance, and high-density mapping found substantially greater correspondence between enhancement regions and unipolar than bipolar low voltage areas [35]. Multimodal substrate data may help distinguish trigger-dominant AF from advanced substrate-dominant disease and inform patient-specific models. Furthermore, digital twin simulation of amiodarone response has already shown proof-of-concept prognostic value after AF ablation [7].

### 5.5. Molecular, Genomic, and Pharmacogenomic Data

Molecular data remains promising but clinically immature. Ion channel and calcium-handling variants may influence drug sensitivity and proarrhythmia, while pharmacokinetic variants may alter metabolism. The chromosome 4q25 variant rs10033464 near *PITX2* has been associated with differential symptomatic response to AAD therapy [27], but single variants are unlikely to provide sufficient predictive value. Future models may integrate polygenic scores, pharmacogenomics, and circulating molecular markers with ECG and clinical data. MicroRNAs represent one candidate molecular layer for such integration. In a spontaneously hypertensive rat model, miR-328 showed diagnostic and predictive potential for AF, illustrating how circulating or tissue-derived molecular signatures could contribute to multimodal phenotyping, although their ability to predict comparative AAD response remains unproven [36].

### 5.6. Machine Learning Approaches

Supervised learning suits defined outcomes such as cardioversion success, recurrence, QT prolongation, toxicity, or discontinuation. Deep learning is most useful for raw ECGs, imaging, electrograms, and other high-dimensional signals. Survival and competing risk models can represent time to recurrence, toxicity, ablation, or death, while longitudinal architectures can update predictions as new data arrive. Causal ML is required when the objective is to estimate outcomes under alternative drugs rather than prognosis under observed care. However, individualized treatment effect methods remain vulnerable to baseline, time-varying, and unmeasured confounding [37]. Reinforcement learning could support sequential dose adjustment or treatment switching, but retrospective policy learning is not evidence that a policy is safe.

Multimodal models can integrate heterogeneous data, while foundation models pretrained on millions of ECGs may reduce task-specific data requirements and enable transfer to drug response applications. *ECGFounder*, for example, was pretrained on more than 10 million ECGs and demonstrated transfer across multiple diagnostic and clinical tasks, although it was not specifically developed for AAD selection [38].

### 5.7. Explainable and Uncertainty-Aware Artificial Intelligence

For treatment selection, discrimination alone is inadequate. Models should provide calibrated probabilities for efficacy and major toxicities, clinically intelligible contributing variables, and uncertainty estimates identifying unreliable recommendations. They should detect out-of-distribution patients whose drug, dose, comorbidity combination, ethnicity, monitoring technology, or care setting was poorly represented during development. Empirical evaluations show that uncertainty methods performing acceptably in distribution may lose coverage under dataset shift [39]. Transparent reporting, external validation, calibration assessment, and explicit handling of missing data and intended use should follow contemporary TRIPOD+AI principles [40]. The clinically appropriate output is not a single drug label, but ranked comparative estimates accompanied by uncertainty and reasons for clinician review (Table 1).

## 6. Artificial Intelligence Across the Antiarrhythmic Drug-Treatment Pathway

**Figure 4:**
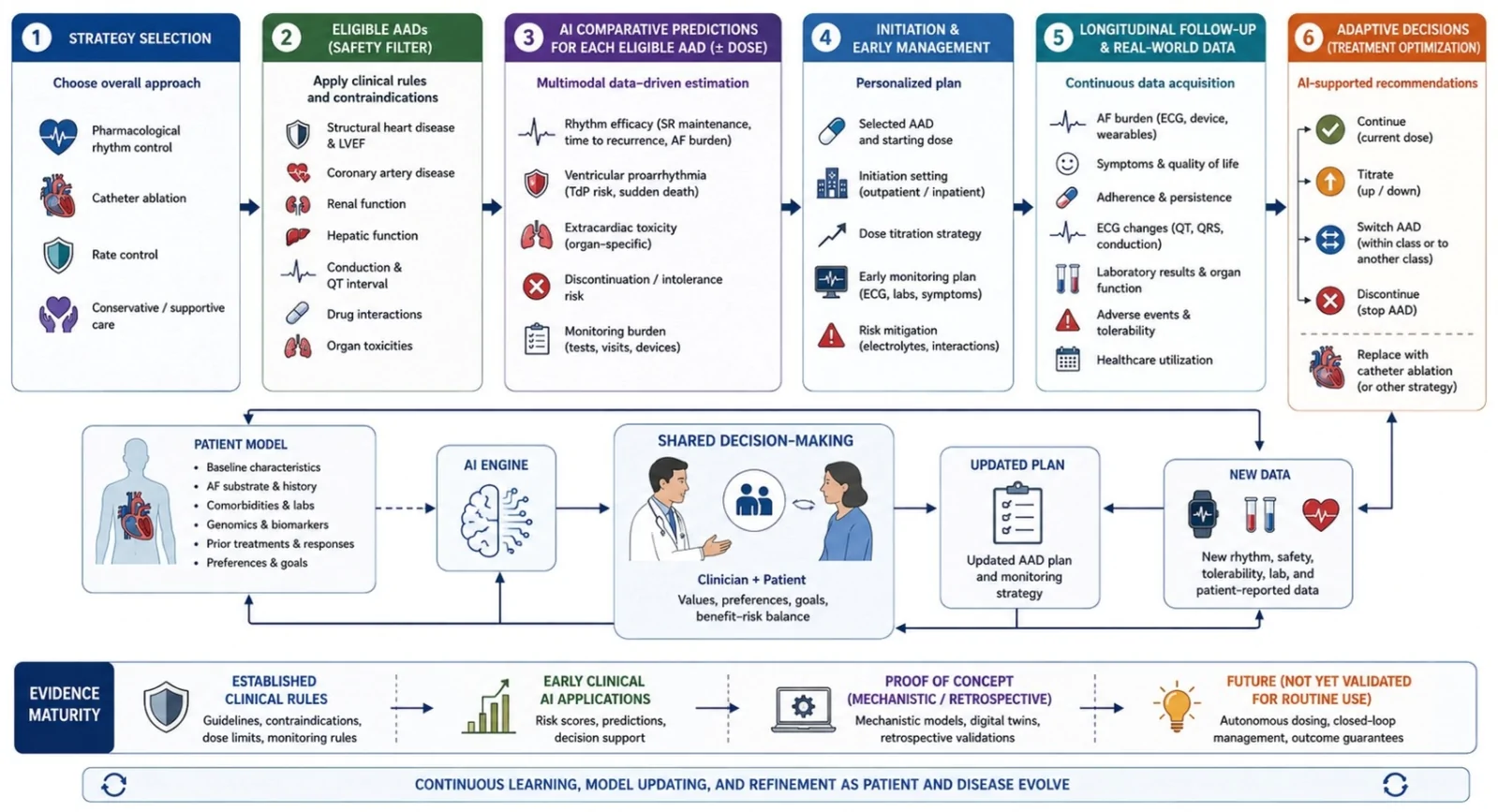
Artificial intelligence (AI) across the antiarrhythmic drug (AAD)-treatment pathway in atrial fibrillation (AF). Six linked stages run from selection between pharmacological rhythm control and alternative strategies, through safety-based eligibility, comparative estimation, individualized dosing, and longitudinal assessment, to adaptive treatment modification. A parallel evidence track indicates the maturity of evidence supporting each stage, from established clinical rules to currently unvalidated capabilities. Feedback arrows show new rhythm and safety data updating the patient model. LVEF—left ventricular ejection fraction; SR—sinus rhythm; TdP—*torsades de pointes*.

The potential value of AI extends across the entire AAD pathway, from deciding whether pharmacological rhythm control is appropriate to recognizing when treatment should be changed or replaced. These decisions are sequential and interdependent: a model that predicts recurrence without considering alternative treatments, toxicity, monitoring requirements, and subsequent disease evolution cannot provide clinically useful therapeutic guidance. Current evidence supports several component applications, particularly prediction of cardioversion outcomes, AF recurrence, QT prolongation, and selected adverse effects. However, no validated AI system currently performs comprehensive, comparative AAD selection in routine AF care. Figure 4 illustrates the potential integration of AI across the longitudinal AAD-treatment pathway.

### 6.1. Identifying Patients Likely to Benefit from Pharmacological Rhythm Control

The first decision is not which AAD to prescribe, but whether pharmacological rhythm control is the most appropriate strategy. Early rhythm control improves cardiovascular outcomes in selected patients with recently diagnosed AF and cardiovascular conditions, but the therapeutic package evaluated in EAST-AFNET 4 included AADs, cardioversion, and ablation rather than an isolated drug strategy [1,2,3]. For an individual patient, the relevant alternatives may include rate control, cardioversion without long-term AAD therapy, early catheter ablation, risk factor and substrate modification, or a combined drug-ablation approach. Catheter ablation provides greater suppression of recurrent AF than drug therapy in many populations, most consistently in symptomatic paroxysmal AF and when the outcome is freedom from documented arrhythmia. However, its invasiveness, availability, procedural risks, and variable durability preclude a uniform first-line solution [3,41].

Artificial intelligence could support this choice by integrating age, symptoms, AF duration and burden, atrial structure, ventricular function, comorbidity trajectories, previous rhythm control attempts, procedural suitability, patient preference, and predicted toxicity. The desired output would be a comparison of expected outcomes under several clinically plausible strategies rather than a probability that a clinician will choose rhythm control. This distinction is important because models trained on observational treatment decisions may learn referral patterns, local practice, or treatment access rather than true treatment benefit. An electronic health record model has shown that ML can reproduce decisions concerning rhythm control strategy in AF, but prediction of observed clinical management does not establish which strategy would have produced the best outcome [42]. AI-based candidacy assessment will therefore require causal rather than merely classificatory reasoning.

A useful system should also recognize that candidacy changes over time. A patient with recent-onset, symptomatic, trigger-dominant AF may initially be well suited to AAD therapy, whereas progressive atrial cardiomyopathy, recurrent hospitalization, intolerable toxicity, or rising AF burden may shift the balance toward ablation. Conversely, substrate modification through weight reduction, sleep apnea treatment, blood pressure control, or heart failure therapy may improve the expected durability of pharmacological rhythm control. Strategy selection should consequently be repeated rather than treated as a one-time baseline decision.

### 6.2. Selecting Among Antiarrhythmic Drugs

Once pharmacological rhythm control is selected, conventional guidelines first exclude unsafe options, as outlined in Section 3.2 [2,3,6], and AI should preserve this safety gate. Its additional purpose would be to rank eligible drugs according to individualized probabilities of rhythm efficacy, proarrhythmia, extracardiac toxicity, discontinuation, and monitoring burden.

### 6.3. Class Ic Drugs

Flecainide and propafenone are effective options in patients without significant structural or ischemic heart disease and can be used for long-term rhythm maintenance or, in carefully selected patients, as a “pill-in-the-pocket” strategy [2,3,6,43]. Selection within this class is nevertheless more complex than the shared Vaughan Williams classification suggests. Relevant factors include baseline conduction, use-dependent QRS widening, sinus and atrioventricular node function, atrial flutter susceptibility, renal and hepatic function, metabolic phenotype, concomitant atrioventricular nodal blockade, and previous response. Propafenone has additional *β*-blocking properties and substantial metabolic variability, whereas flecainide exposure is affected by renal function and drug interactions [6].

Mechanistic AI and computational models may be particularly valuable for sodium channel blockers because their effects depend strongly on activation rate, conduction reserve, atrial geometry, and ionic phenotype. In silico populations of this kind are constructed by repeatedly sampling the parameters of a cellular electrophysiological model, most often the conductances of the principal ionic currents, and retaining only those parameters whose simulated behavior falls within experimentally observed ranges. In such a population of atrial electrophysiological models, simulated flecainide exposure was antiarrhythmic in most virtual phenotypes, but proarrhythmic behavior emerged in a subset, and tissue dilation reduced successful AF suppression [44]. ML analysis identified combinations of ionic and tissue properties associated with divergent drug responses. Such findings demonstrate why a population average drug effect cannot be assumed to apply uniformly. However, simulated response cannot yet substitute for clinical outcome prediction, because virtual populations incompletely represent fibrosis, pharmacokinetics, autonomic influences, adherence, and extracardiac toxicity. However, clinical usefulness requires patient-level calibration, prospective comparison of simulated and observed responses, and added value beyond clinical and ECG predictors.

A clinically useful class Ic model would combine baseline and exercise-related QRS behavior, atrial substrate, AF episode characteristics, organ function, concomitant therapy, and previous drug exposure. It should predict not only recurrence but also the risk that therapeutic concentrations produce excessive conduction slowing or organize AF into rapidly conducted atrial flutter. Serial ECG changes following initiation may be more informative than baseline measurements alone.

### 6.4. Class III Drugs

Selection among amiodarone, dronedarone, sotalol, dofetilide, and other geographically available class III agents requires a different balance of efficacy and toxicity. Amiodarone is generally highly effective for maintenance of sinus rhythm and remains the preferred option in patients with heart failure with reduced ejection fraction and in significant structural heart disease. However, its cumulative thyroid, pulmonary, hepatic, neurological, ocular, dermatological, and bradyarrhythmic toxicity restricts its desirability as a default long-term option [2,3,6]. Dronedarone is less effective than amiodarone for maintaining sinus rhythm but avoids iodine-related thyroid toxicity and some cumulative organ effects; it is contraindicated in permanent AF and in patients with NYHA class III-IV or recently decompensated heart failure [12,45]. Sotalol and dofetilide require close attention to renal elimination, QT prolongation, bradycardia, electrolyte status, and concomitant repolarization-prolonging drugs [2,3,6].

The relevant AI task is therefore multi-outcome comparison. A patient with a high probability of recurrence on dronedarone but substantial predicted amiodarone pulmonary toxicity may have no clearly dominant drug. Another patient may have relatively low extracardiac risk but limited repolarization reserve, making amiodarone preferable to a more torsadogenic alternative despite its long-term toxicity. A single recurrence score cannot capture these trade-offs. They require separate, calibrated estimates of rhythm efficacy, ventricular safety, organ-specific toxicity, treatment discontinuation, and the time horizon over which benefit is sought.

Drug ranking should also account for the therapeutic objective. Short-term stabilization after cardioversion, suppression during the post-ablation healing period, and indefinite rhythm maintenance require different weighting of delayed cumulative toxicity. A model that ignores intended treatment duration may recommend an agent with an inappropriate benefit-risk profile even if its prediction of short-term rhythm efficacy is accurate.

### 6.5. Comparative Treatment Selection

Most existing prediction studies estimate the risk of an outcome among patients who received one observed treatment. Such models are prognostic, not treatment comparative. A patient predicted to have a 60% recurrence risk on amiodarone cannot be assumed to fare better on flecainide, dronedarone, or ablation unless counterfactual outcomes under those alternatives are estimated.

At present, comparative AI-supported AAD selection remains largely aspirational. The literature contains prediction of observed strategy, recurrence under specific treatment contexts, and simulated drug response, but little prospective estimation of patient-level outcomes under several eligible AADs. Section 8.2 discusses the required methods and the conditions under which they remain valid.

### 6.6. Predicting Pharmacological Cardioversion Success

Pharmacological cardioversion provides a relatively well-defined AI target because the outcome occurs over a short interval. Relevant predictors include AF duration, previous episodes, atrial dimensions and function, ventricular rate, surface ECG characteristics, autonomic state, structural heart disease, electrolytes, inflammatory and metabolic variables, and the selected drug and dose. Nevertheless, even apparently simple endpoints require precise definition: conversion within 30 min, several hours, or 24 h represent different therapeutic questions, and spontaneous conversion must be distinguished from drug-attributable response.

An ML analysis of patients referred for elective electrical cardioversion modeled several linked outcomes, including spontaneous restoration of sinus rhythm, pharmacological conversion before the procedure, immediate electrical cardioversion success, six-month recurrence, and maintenance of rhythm control [46]. The study illustrates how demographic, clinical, biochemical, echocardiographic, treatment, and procedural data can be integrated across a cardioversion pathway. However, the analysis was retrospective and single-center, based on the electronic health records of 429 consecutive patients referred for elective cardioversion, and modeled the observed care pathway rather than randomized allocation to alternative agents [46]. Future models should be drug-specific, time-specific, and calibrated across recent-onset and persistent AF populations. They should also indicate when waiting for spontaneous conversion, proceeding directly to electrical cardioversion, or selecting a different pharmacological agent is more appropriate.

### 6.7. Predicting Maintenance of Sinus Rhythm

Maintenance of sinus rhythm represents several distinct prediction settings. After spontaneous or pharmacological conversion, recurrence may reflect unresolved triggers or an inadequately suppressed substrate. After electrical cardioversion, early recurrence may reflect transient electrical remodeling, whereas later recurrence more strongly reflects atrial cardiomyopathy and comorbidity progression. Following initiation of long-term AAD therapy, outcomes depend on exposure, adherence, dose attainment, and toxicity. After catheter ablation, pulmonary vein reconnection, residual extra-pulmonary substrate, and post-procedural inflammation alter both recurrence mechanisms and drug effects.

Machine learning models used by Kwon and collaborators, combining clinical and ECG information, have achieved modest prediction of AF recurrence after electrical cardioversion, with combined inputs generally performing better than either data type alone [47]. Discrimination was low, with areas under the curve (AUC) of 0.57 for clinical features alone, 0.60 for ECG alone, and 0.63 for both combined, with ten-fold cross-validation and no external validation. At the reported operating point, sensitivity was 84.7% but specificity only 28.2%, so most patients would be classified as being at risk regardless of outcome. Their limited discrimination and need for external validation highlight the difficulty of predicting a dynamic substrate from a single baseline snapshot. Serial measurements may offer greater value: early ectopy, atrial conduction changes, short recurrent episodes, QT or QRS response, and evolving AF burden could update recurrence estimates after therapy begins.

Another consideration is treatment duration. The *Flecainide Short-Long* (Flec-SL) trial showed that short-term flecainide after cardioversion prevented some early recurrences, but continued treatment was more effective over the longer treatment interval [48]. After ablation, short-term AAD administration can reduce early atrial arrhythmias without consistently preventing late recurrence after withdrawal, whereas continued AAD therapy beyond the blanking period may benefit selected patients whose previously ineffective drug becomes effective after pulmonary vein isolation [13,15]. AI models should therefore distinguish immediate stabilization, blanking period suppression, and durable maintenance rather than collapsing them into a single recurrence outcome.

### 6.8. Predicting Changes in Atrial Fibrillation Burden Rather than Binary Recurrence

Time to first documented recurrence is convenient but clinically incomplete. An AAD may reduce episode frequency, duration, or ventricular rate without eliminating AF, and guidelines recognize that less frequent, briefer, or less symptomatic recurrences may constitute meaningful therapeutic benefit. AF burden is thus a patient-relevant measure of therapeutic benefit rather than a formally validated surrogate endpoint for cardiovascular outcomes [2]. Continuous monitoring data from ablation trials similarly show that treatment effects can be expressed as substantial reductions in AF burden even when occasional recurrence remains detectable [41]. Comparable evidence now exists for pharmacological therapy. In a multicenter retrospective analysis of 128 patients with cardiac implantable electronic devices, median AF burden fell from 5.9% [1.3–29.7%] before AAD initiation to 0.1% [0–1.5%] at final interrogation, a median relative reduction of 97.4% [IQR 35.7–100%], consistent across amiodarone and non-amiodarone agents and across high (≥90%) and low (≤20%) baseline burden. Among patients who still had AF exceeding 30 s after initiation, the median reduction was 72.1% [5.3–96.9%]. Consequently, under a binary endpoint, these patients would be classified as treatment failures despite a substantial measured effect [49].

Artificial intelligence can model AF burden as a continuous and longitudinal outcome, allowing prediction of absolute burden reduction, episode clustering, time spent in rapid AF, and symptom-rhythm concordance. This approach may identify partial responders who benefit from continued therapy and patients whose apparently infrequent recurrence conceals prolonged asymptomatic episodes. It also creates methodological challenges: monitoring intensity differs among patients, wearable data are incomplete, and photoplethysmographic detection may be less reliable during activity. Models should distinguish absence of detected AF from absence of monitoring and should report the monitoring technology and analyzable observation time.

### 6.9. Personalizing Dose and Drug Initiation Strategies

Dose selection influences both efficacy and toxicity but is rarely treated as a predictive problem. Starting dose, escalation rate, renal adjustment, inpatient versus outpatient initiation, and surveillance intensity are generally determined by drug labeling and broad clinical categories [2,3,6]. AI could refine these decisions by estimating individual exposure and electrophysiological response from age, body composition, renal and hepatic function, interacting medications, electrolytes, baseline ECG, and previous dose–response data.

The immediate clinical opportunity is not autonomous dosing but risk-adapted initiation. Patients predicted to develop marked QT prolongation, bradycardia, pauses, or QRS widening could undergo inpatient initiation or intensified ECG surveillance, while appropriately selected lower-risk patients might avoid unnecessary hospitalization where regulations permit. Serial post-dose ECG and laboratory data could update the model after each exposure. Such systems must remain bounded by approved dosing rules and should never infer safety from the absence of adverse events in poorly monitored patients.

### 6.10. Predicting Repolarization Risk and Ventricular Proarrhythmia

Ventricular safety is among the most mature AI applications relevant to AAD therapy, although the outcomes predicted are predominantly repolarization measures rather than arrhythmic events. ML models using electronic health record variables have predicted drug-induced long QT susceptibility, while deep learning analysis of the 12-lead ECG has identified signatures associated with drug exposure, congenital or acquired long QT states, and drug-induced *torsades de pointes* [31,32]. The endpoint in Prifti’s study was identifying sotalol exposure in 1029 healthy volunteers, in whom the network outperformed QTc (ROC-AUC 0.98 versus 0.72, *p* < 0.001), along with classifying 487 patients with congenital long QT syndrome and 48 patients with previously documented drug-induced *torsades de pointes*. No cited model has prospectively predicted adjudicated *torsades de pointes*, and none has been evaluated in a population with AF, but these approaches may capture repolarization vulnerability not adequately represented by a single QTc measurement.

Continuous surveillance may further improve risk assessment. A deep learning system developed to reconstruct QT and QTc intervals from continuously recorded single-lead implantable monitor data identified high-risk prolongation after initiation of class III AADs [33]. Prolonged outpatient exposure was associated with a more than fourfold increase in ventricular arrhythmia events in a cohort of 1676 outpatients (3.97% versus 0.86%; adjusted odds ratio 4.24, 95% CI 1.81–9.90) and an AUC of 0.94 [33]. This suggests that risk may emerge outside the short inpatient observation window and that cumulative duration above a dangerous threshold could be more informative than the maximum QTc alone.

Mechanistic emulators provide a complementary approach. Sex-specific cardiac emulators integrating blockade of multiple ion channels with three-dimensional electrophysiology have reproduced QT prolongation and arrhythmic behavior across virtual male and female phenotypes with substantially lower computational cost than full simulations [50]. Such models could eventually compare anticipated electrophysiological effects of candidate drugs and doses. However, they remain predominantly preclinical and do not yet integrate the complete clinical context of AF, including dynamic electrolytes, bradycardia, organ dysfunction, interacting drugs, or adherence. Prediction of conduction slowing, ventricular tachyarrhythmias, bradyarrhythmias, and *torsades* should therefore combine mechanistic and clinical data rather than rely on either alone.

Machine learning models using electronic health record data have predicted severe drug-induced QT prolongation [51]. Recently, in a retrospective health-system cohort, a gradient-boosted model predicting a QTc of 500 ms or more within one year outperformed the RISQ-PATH and Tisdale scores (AUROC 0.859 versus 0.701 overall, and 0.855 versus 0.770 in a predominantly inpatient subgroup of 110,558 patients with an 8.8% event rate), with internal cross-validation only and no AF-specific analysis [51].

Another AF-specific application is AI-based QT correction during AF, where conventional formulas are unreliable. Specifically, a convolutional neural network applied to ECGs recorded during AF estimated the corresponding sinus rhythm QTc interval, an important intermediate marker of susceptibility to *torsades de pointes*, achieving a mean absolute error of 22.2 milliseconds and outperforming conventional correction methods when ruling out QTc prolongation. However, it predicted QTc rather than *torsades de pointes*, sudden cardiac death, or another clinical toxicity endpoint, so its clinical value remains primarily as a risk stratification and monitoring aid [52]. The field therefore still requires prospective, externally validated studies in AF rhythm control cohorts integrating ECG waveforms, renal function, electrolytes, comorbidities, drug exposure, and genetic risk.

### 6.11. Predicting Extracardiac Toxicity

Extracardiac adverse effects frequently determine whether an effective AAD can be continued. Amiodarone is the principal example because its long half-life, tissue accumulation, and multiorgan toxicity create a dynamic risk that may rise with cumulative exposure [6]. Explainable ML models have predicted amiodarone-associated thyroid dysfunction using longitudinal clinical data, with external validation and modifiable decision thresholds intended to support different screening priorities [53]. Specifically, the external validation included 2422 patients at a separate hospital, among whom 11.4% developed thyroid dysfunction, distinguishing this work from most models discussed here. The choice of threshold matters because discrimination and precision diverge: at the reported operating point, discrimination was high (AUROC 0.934) while precision was 0.632, so a considerable share of flagged patients would not develop thyroid dysfunction. The model therefore identifies patients who warrant closer thyroid surveillance under amiodarone; it does not estimate the corresponding risk under dronedarone or sotalol. Models of this kind demonstrate the feasibility of moving from uniform surveillance intervals toward risk-adapted monitoring.

Analogous models could address pulmonary, hepatic, neurological, ocular, and dermatological toxicity, but evidence is less mature and is often derived from general drug-safety or pharmacovigilance datasets rather than AF-specific populations. Relevant predictors may include age, pre-existing pulmonary or thyroid disease, cumulative dose, organ function, concomitant medication, symptoms, imaging, and serial laboratory changes [2,3,6]. Rare outcomes create substantial class imbalance, and diagnostic labels may be uncertain because toxicity is often a diagnosis of exclusion. Models should therefore support earlier investigation rather than independently diagnose organ injury.

### 6.12. Detecting Drug–Drug and Drug–Disease Interactions

Multimorbidity and polypharmacy make AAD safety context-dependent. Renal deterioration can increase exposure to sotalol or dofetilide; electrolyte loss from diuretics can magnify *torsades* risk; bradycardia from combined nodal blocking therapy can increase reverse-use-dependent QT prolongation; and cytochrome-mediated interactions can alter concentrations of several AADs [2,3,6]. Non-cardiovascular medications may add QT prolongation, conduction slowing, hepatic toxicity, or pharmacokinetic inhibition.

Conventional interaction databases generate numerous alerts but often fail to rank them according to patient-specific clinical importance. AI could combine the interacting drug pair with dose, organ function, electrolytes, baseline and serial ECGs, and previous tolerance to estimate an individualized hazard. However, training labels derived from alert overrides or coded adverse events may reproduce documentation bias. Interaction models should therefore explain the predicted mechanism, distinguish pharmacokinetic from pharmacodynamic risk, and identify which modifiable factor (drug withdrawal, dose reduction, electrolyte correction, or surveillance) would most effectively reduce risk.

### 6.13. Artificial Intelligence-Supported Longitudinal Monitoring

The value of AI may be greatest after treatment begins. Serial ECGs can reveal QT or QRS changes, bradycardia, atrial flutter, and pharmacodynamic response. Wearables and implanted devices can quantify AF burden and ventricular rate patterns. Laboratory data can detect renal, hepatic, thyroid, or electrolyte changes, while symptoms, activity, sleep, adherence, adverse event reports, and hospitalization history add clinical context.

A longitudinal model should compare each patient with both the relevant population and their own pretreatment state. Within-patient deviation may identify emerging toxicity or loss of efficacy earlier than a fixed threshold. Monitoring intensity could be adapted according to current risk, but reduced surveillance must not become self-reinforcing: patients monitored less frequently will appear to have fewer detected abnormalities. Data provenance, missingness, device changes, and care received outside the health system must therefore be represented explicitly.

### 6.14. Adaptive Treatment Modification

The ultimate objective is a continuously updated therapeutic model. At each reassessment, new rhythm, ECG, laboratory, symptom, adherence, organ-function, and comorbidity data would revise the expected benefit and risk of continuing the current drug, changing the dose, switching agents, discontinuing treatment, or proceeding to ablation. The system should also recognize changes in patient priorities, such as willingness to accept monitoring or invasive treatment.

This model resembles a dynamic treatment regime rather than a one-time prediction. Reinforcement learning and other sequential decision methods could theoretically identify policies that maximize long-term rhythm control while minimizing toxicity and burden. Yet policies learned retrospectively may favor actions that reflect historical access, clinician selection, or incomplete monitoring rather than optimal care. Prospective evaluation should therefore proceed through silent validation, clinician-facing decision support, controlled impact assessment, and randomized trials reported according to AI-specific clinical trial standards [37,54]. Until such evidence exists, AI should function as an uncertainty-aware comparative assistant under clinician oversight, not as an autonomous prescriber.

## 7. Computational Electrophysiology and Digital Twins for Virtual Drug Testing

**Figure 5:**
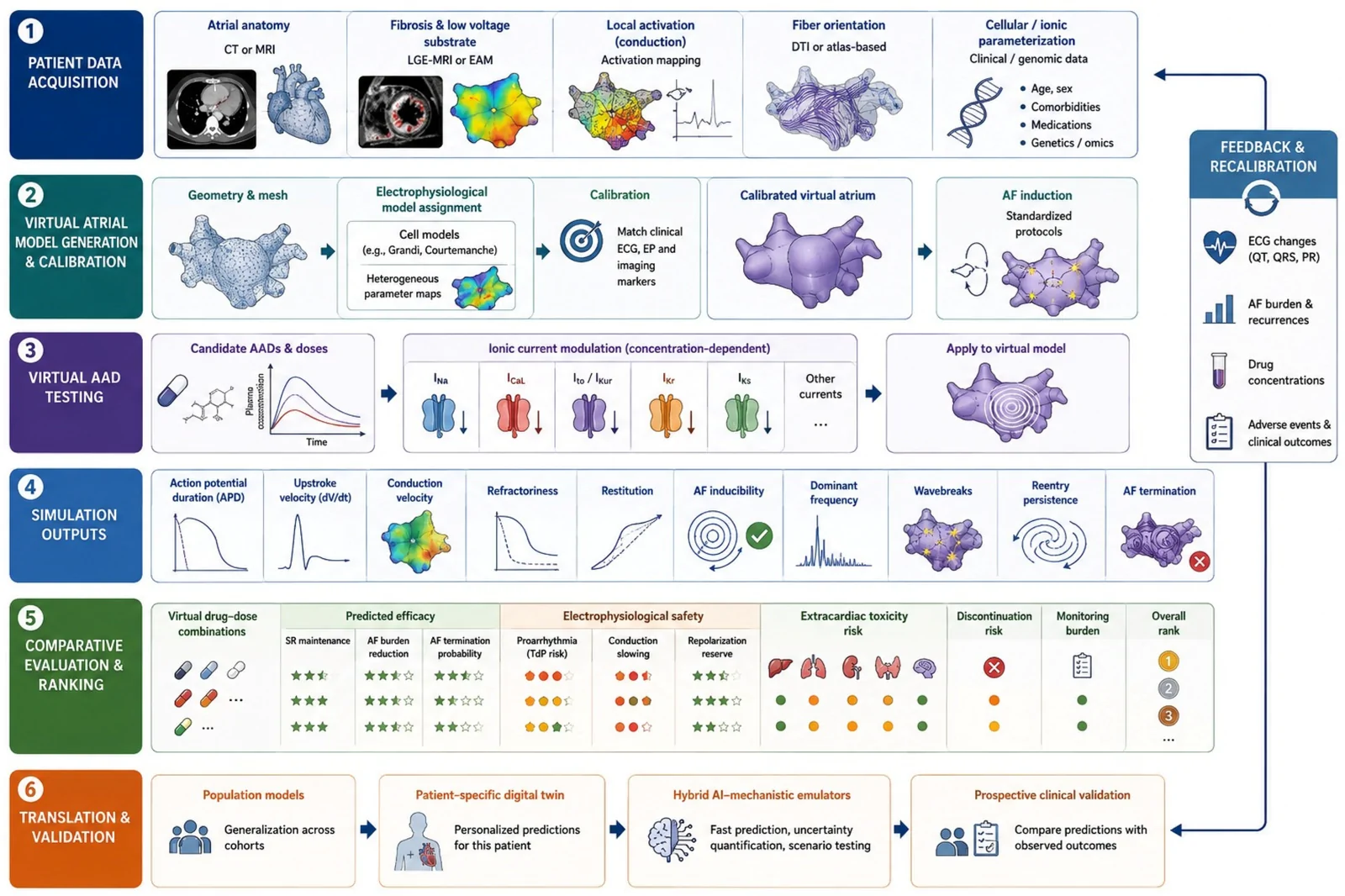
From patient data to virtual antiarrhythmic drug (AAD) testing in an atrial digital twin. A sequential workflow proceeds from patient-specific imaging, mapping, and clinical data to a calibrated virtual atrium in which atrial fibrillation is induced under standardized protocols. Candidate drugs and doses are applied through concentration-dependent modification of ionic currents, and simulation outputs rank drug–dose combinations by predicted efficacy and electrophysiological safety. A final layer distinguishes population models, patient-specific digital twins, hybrid emulators, and prospective clinical validation; only the first three have been demonstrated. CT—computed tomography; DTI—diffusion tensor imaging; dt—delta time; dV—delta amplitude; EAM—electro-anatomical mapping; EP—electrophysiological; I_CaL_—L-type calcium current; I_Kr_—rapid component of the delayed rectifying potassium current; I_Ks_—slow component of the delayed rectifying potassium current; I_Kur_—ultrarapid component of the delayed rectifying potassium current; I_Na_—voltage-gated sodium current; I_to_—transient outward potassium current; LGE—late gadolinium enhancement; MRI—magnetic resonance imaging; SR—sinus rhythm; TdP—*torsades de pointes*.

Computational electrophysiology differs from conventional data-driven prediction. Instead of learning associations alone, it represents the mechanisms through which electrical activation propagates, AF is initiated and maintained, and AADs modify cellular and tissue behavior. A digital twin extends this approach by calibrating the virtual system to an individual patient and, ideally, updating it as new data emerge. Figure 5 outlines the steps involved, from patient-specific imaging and mapping to ranking drug–dose combinations, and distinguishes what has been demonstrated from what remains hypothetical.

### 7.1. From Population Models to Patient-Specific Virtual Atria

Population models generate cohorts of virtual atrial phenotypes by varying ionic conductances, action potential properties, conduction parameters, anatomy, or tissue dimensions within biologically plausible ranges. They help identify determinants of heterogeneous drug response and enable in silico trials at otherwise impractical scales. Simulations across 100 ionic profiles, 200 AF episodes, and 10 pharmacological interventions have identified excitability and restitution properties associated with AF sustainability and pharmacological cardioversion [55].

Patient-specific models add constraints derived from cardiac computed tomography or magnetic resonance imaging, electro-anatomical voltage and activation maps, and clinical electrograms. A virtual atrium may incorporate chamber geometry, pulmonary veins, fibrosis or low voltage tissue, fiber orientation, anisotropic conduction, local activation times, and cellular ionic current formulations. Lim et al. integrated individual left atrial anatomy, bipolar voltage, estimated fibrosis, fiber orientation, and activation data into realistic AF simulations [56] and emphasize that the result is not a complete biological replica, but a context-specific representation of clinically relevant electrical behavior.

### 7.2. Simulating Antiarrhythmic Drug Effects

Drug action is introduced by modifying mathematical representations of sodium, potassium, calcium, and other ionic currents according to concentration-dependent channel blockade. These cellular changes alter action potential duration, upstroke velocity, refractoriness, restitution, excitability, and conduction velocity. Tissue-scale simulations can then assess AF inducibility, dominant frequency, wavebreak, reentry organization, and termination.

Such models expose interactions hidden by population averages. Sodium channel blockade may suppress rapid reentry by reducing excitability, yet excessive conduction slowing may stabilize reentry in susceptible tissue. Population model studies have identified electrophysiological profiles in which flecainide was antiarrhythmic or proarrhythmic [44], while genotype-informed simulations have demonstrated different responses to amiodarone, sotalol, dronedarone, flecainide, and propafenone under wild-type and *PITX2*-deficient conditions [57].

### 7.3. Virtual Comparison of Drugs and Doses

The clinically compelling concept is to treat the virtual patient before treating the biological patient. Several eligible drugs and concentrations could be applied to the same calibrated model, allowing comparison of AF termination, residual vulnerability, conduction slowing, and potential safety signals. Because each option is tested against an otherwise identical virtual substrate, this approach more closely approximates a within-patient comparison than conventional prognostic modeling. In silico trials have already compared multiple rhythm control agents across heterogeneous virtual atrial phenotypes and identified treatment-specific differences in predicted cardioversion success [7,55]. Nevertheless, virtual rankings remain hypothesis-generating until prospectively linked to measured drug exposure and clinical outcomes.

### 7.4. Digital-Twin Prediction of Amiodarone Response

The strongest clinical proof of concept was reported in 2024. Patient-specific left-atrial digital twins were constructed from computed tomography anatomy and electro-anatomical mapping in 115 patients receiving amiodarone after AF ablation [7]. The models incorporated fibrosis, fiber orientation, voltage, and local activation data and simulated AF under increasing amiodarone concentrations. Higher concentrations prolonged action potential duration, reduced peak upstroke velocity, and increased virtual AF termination. Virtual responders had lower one-year AF or atrial tachycardia recurrence than non-responders: 20.8% versus 45.1% [7]. However, that difference was based on 24 of 115 patients classified as virtual responders, among whom five recurrences occurred, and the confidence interval for the adjusted hazard ratio approached unity (0.37, CI 0.14–0.98) [7]. Moreover, the study was single-center and retrospective, involved a selected post-ablation population, used a left atrial monolayer, and evaluated only amiodarone. The virtual ablation lesions were also not individualized to each patient’s actual procedure, and prospective randomized validation remains necessary [7]. Despite all these limitations, the study emphasizes the potential utility of digital twins to guide an amiodarone-based personalized approach.

### 7.5. Hybrid Mechanistic-Artificial Intelligence Models

Machine learning can make digital twins more scalable by approximating computationally expensive simulations, identifying influential parameters, calibrating models to sparse measurements, and updating predictions from longitudinal ECG, imaging, rhythm, or drug response data. Cardiac emulators have reproduced complex three-dimensional drug-induced QT responses at substantially lower computational cost than full simulations, including sex-specific differences in repolarization and arrhythmic behavior [50]. Hybrid systems may therefore retain mechanistic constraints while gaining the speed required for bedside comparison, uncertainty analysis, and repeated recalibration. However, this conceptual architecture should not be mistaken for an integrated model. The atrial and ventricular domains discussed here currently rely on different datasets, model classes, and validation paradigms. Atrial digital twins are built from atrial anatomy and are evaluated against atrial outcomes, such as AF termination or recurrence. In contrast, the ventricular emulators described above are based on ventricular electrophysiology and are evaluated primarily against repolarization-related endpoints, largely in preclinical settings. An atrial digital twin may therefore indicate whether a drug is likely to terminate AF while providing little direct information about that patient’s risk of *torsades de pointes* or ventricular fibrillation, unless it is linked to a separately validated ventricular model. A whole-patient benefit–risk model would need to integrate these domains; however, placing them within a single conceptual architecture, as in Figure 5, does not imply that an integrated and jointly validated model currently exists.

### 7.6. Current Limitations

Important barriers remain. Many atrial twins omit the right atrium, interatrial conduction, wall thickness, autonomic effects, and spatially heterogeneous pharmacokinetics. Fibrosis and fiber orientation are estimated indirectly, while different parameter combinations may reproduce the same activation pattern, creating identifiability uncertainty. Construction may depend on invasive mapping; computation remains demanding; and outputs are sensitive to ionic models, drug-channel assumptions, induction protocols, and modeled ablation lesions.

Most importantly, technical plausibility and retrospective association do not establish clinical utility. Digital twins require reproducibility, calibrated uncertainty, prospective validation, and evidence of improved treatment outcomes before virtual drug testing can support routine prescribing.

## 8. From Algorithmic Prediction to Clinical Decision Support

### 8.1. Prediction Is Not Equivalent to Treatment Recommendation

A prognostic model estimates what is likely to happen under a specified, or sometimes poorly specified, pattern of care. It may predict AF recurrence, QT prolongation, treatment discontinuation, or hospitalization, yet still provide no evidence that changing treatment will improve that outcome. A patient classified as being at high risk of recurrence while receiving amiodarone cannot be assumed to benefit from flecainide, dronedarone, sotalol, or ablation. The prediction may simply identify advanced atrial disease that confers poor outcomes under every strategy. Models developed from routine care may also learn prescribing patterns, treatment access, or contraindications rather than true therapeutic benefit. Decision support must therefore distinguish prediction under observed management from estimation of the effect of choosing one intervention instead of another [37,58,59].

### 8.2. Comparative and Causal Treatment Effect Estimation

The clinically relevant question is counterfactual: what would happen to the same patient under each plausible AAD, dose, or alternative rhythm control strategy? Because only one outcome is observed, individualized treatment effect models must estimate the unobserved alternatives. Randomized trials provide the strongest protection against confounding, but they are usually powered for average treatment effects and may contain too few events for reliable individual-level estimation. Causal ML methods can explore heterogeneous effects in trial data or observational cohorts, but validity depends on treatment positivity, consistent outcome definitions, adequate confounder adjustment, and cautious handling of time-varying and unmeasured confounding [37,58,59,60].

For AF, comparative models should estimate several outcomes separately: maintenance of sinus rhythm or reduction in AF burden, proarrhythmia, organ-specific toxicity, treatment discontinuation, monitoring burden, and subsequent ablation. Target trial emulation may supplement randomized evidence, but it cannot remove bias from unmeasured determinants of treatment choice. Outputs should therefore be presented as estimated comparative benefit-risk profiles rather than individualized causal truths.

### 8.3. Proposed Artificial Intelligence-Supported Decision Architecture

A clinically useful architecture should operate in layers. A rule-based safety gate should apply guideline-defined contraindications, dose restrictions, and clinically significant interactions. Drug-specific models should estimate efficacy and major cardiac and extracardiac harms for every eligible option. The system should quantify monitoring intensity, treatment burden, and discontinuation risk. It should report calibration, uncertainty, missing data limitations, and whether the patient lies outside the development population. Finally, it should present a ranked set of options with the principal factors supporting or opposing each choice, rather than issue a single opaque prescription.

Performance should be evaluated beyond discrimination. Calibration, clinically relevant thresholds, net benefit, subgroup performance, external evaluation, and failure analysis are essential. TRIPOD+AI provides contemporary guidance for transparent reporting of prediction-model development and evaluation, but reporting quality alone does not establish clinical utility [40].

### 8.4. Human-Artificial Intelligence Collaboration

The clinician should remain responsible for interpreting estimates, verifying data quality, identifying circumstances absent from the model, and determining whether uncertainty is clinically acceptable. Patient preferences may change the ranking substantially: one patient may prioritize maximal rhythm efficacy, whereas another may reject hospitalization, frequent testing, cumulative toxicity, or invasive treatment. Shared decision-making and dynamic reassessment are integral to contemporary AF management and cannot be reduced to an algorithmic utility score [2].

**Figure 6:**
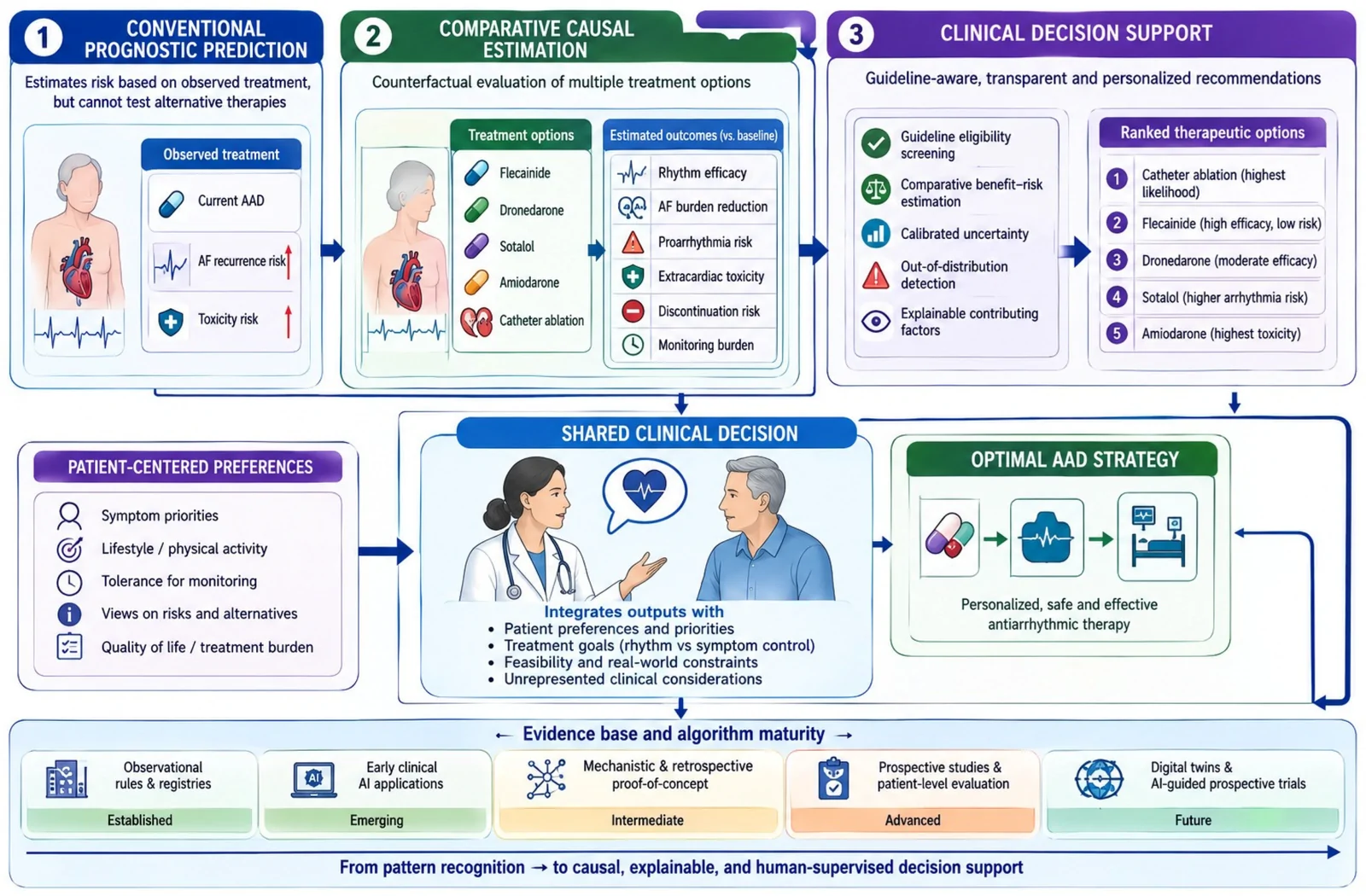
From prognostic prediction to causal, explainable, and human-supervised antiarrhythmic decision support. Three levels of algorithmic output are contrasted: prognostic prediction under observed treatment; comparative causal estimation, in which the same patient is evaluated under several counterfactual options with separate estimates for each outcome domain; and clinical decision support, in which eligibility screening, calibrated uncertainty, out-of-distribution detection, and explainable contributing factors produce a ranked set of options. A central clinician-patient node integrates these with preferences and unrepresented circumstances. AAD—antiarrhythmic drug; AI—artificial intelligence.

The interface should encourage verification rather than automation bias. It should display alternative options, uncertainty, reasons for model abstention, and the anticipated consequences of accepting or overriding a recommendation. Early clinical evaluation must examine human factors, workflow effects, error cases, and unintended consequences, as DECIDE-AI emphasizes [61]. Figure 6 sets out this progression and shows that the three levels differ not in accuracy but in the questions they answer: what will happen, what would happen under each alternative, and what should be presented to a clinician and patient deciding together.

### 8.5. Integration into the Clinical Workflow

At the initial rhythm control consultation, decision support could define eligible strategies and compare drugs. At cardioversion, it could estimate conversion and early recurrence probabilities and guide post-procedure therapy. At hospital discharge, it could verify dose, interactions, organ function requirements, and surveillance. After ablation, it could inform short-term or continued AAD use. During outpatient reassessment, new ECG, AF-burden, laboratory, symptom, adherence, and adverse event data could update the ranking.

Deployment should progress from retrospective development to silent prospective evaluation, clinician-facing feasibility studies, and randomized clinical-impact trials. DECIDE-AI addresses early clinical evaluation, whereas CONSORT-AI specifies transparent reporting of trials involving AI interventions, including intended use, workflow integration, human-AI interaction, and error analysis [54,61]. Until such studies demonstrate improvement in patient-important outcomes, AI should structure and clarify decisions, not replace clinical judgment.

## 9. Methodological, Ethical, Regulatory, and Implementation Challenges

### 9.1. Limited and Fragmented Evidence

Artificial intelligence applications in AF are considerably more mature for rhythm detection, screening, recurrence prediction, and catheter-ablation support than for comparative AAD selection. Published treatment-related studies remain dominated by retrospective, single-center, and proof-of-concept work. Broad reviews describe AI-supported treatment optimization as promising but also emphasize the scarcity of prospective validation and the absence of randomized evidence showing that AI-guided AAD selection improves patient outcomes [62]. Consequently, evidence from adjacent applications, such as general drug-induced QT prediction or organ-toxicity surveillance, must not be presented as direct validation of AF-specific prescribing systems.

### 9.2. Confounding by Indication

Routine care data do not approximate random treatment allocation. Amiodarone is often prescribed to patients with more advanced structural disease, heart failure, or previous treatment failure, whereas class Ic agents are preferentially used in patients with fewer contraindications. Treatment choice also reflects clinician experience, drug availability, patient preference, and access to ablation. A model may therefore associate a drug with poor outcomes because it was selected for higher-risk patients rather than because the drug caused those outcomes. Target trial emulation and causal ML can reduce design-related biases, but they cannot eliminate unmeasured confounding or poor exposure ascertainment [63].

### 9.3. Inconsistent Definitions of Treatment Success

Antiarrhythmic drug studies use heterogeneous and non-interchangeable endpoints, ranging from acute conversion to AF burden, symptom improvement, hospitalization, and discontinuation (Table 2). Time to first recurrence is strongly influenced by monitoring intensity and does not distinguish a brief asymptomatic episode from recurrent prolonged AF. Therefore, AF burden has been proposed as the proportion of monitored time spent in AF, ideally accompanied by the longest continuous episode, but measurement remains dependent on monitoring technology and analyzable observation time [64]. Models trained using incompatible endpoints cannot be directly compared or safely combined.

### 9.4. Dynamic and Competing Outcomes

Efficacy and toxicity evolve simultaneously. Recurrence may prompt dose escalation, switching, cardioversion, or ablation. Adverse effects may reduce adherence, and treatment discontinuation may precede the outcome of interest. Ablation and death can prevent observation of subsequent drug response, while hospitalization may act as both an outcome and a trigger for treatment modification. Static baseline models do not adequately represent these processes. Longitudinal, multistate, competing risk, and joint models are more appropriate, but require precise time-stamped exposure, monitoring, and outcome data.

### 9.5. Dataset Shift and Generalizability

Performance may deteriorate when a model is transferred across institutions, countries, recording technologies, or populations with different prescribing practices and drug availability. External validation should therefore evaluate the locked model in genuinely independent settings and assess discrimination, calibration, clinical thresholds, and subgroup performance. TRIPOD+AI and PROBAST+AI emphasize transparent reporting, applicability, and risk of bias assessment, but adherence to reporting standards does not establish transportability [40,65].

### 9.6. Bias, Fairness, and Under-Representation

Several groups listed in Table 2 may be under-represented or incompletely characterized in development datasets. These groups are particularly relevant to AAD safety because sex, age, renal function, polypharmacy, and comorbidity affect repolarization, drug clearance, and adverse event risk. Wearable-dependent models may further disadvantage patients with limited digital access, low device adherence, or reduced technical literacy. Fairness cannot be reduced to similar AUC values across groups; it also requires equitable access, clinically appropriate thresholds, representative outcomes, and monitoring for differential harm after deployment [66,67].

### 9.7. Explainability, Calibration, and Uncertainty

A highly discriminative model may still provide systematically inaccurate probabilities. Because prescribing depends on absolute benefit-risk estimates, calibration is essential. Systems should also quantify uncertainty, identify missing or unreliable inputs, and abstain when patients are outside the model’s supported population. Explanations should identify clinically intelligible factors influencing each drug-specific estimate, while avoiding the false reassurance that a *post hoc* feature attribution necessarily explains causal reasoning. FUTURE-AI recommends fairness, universality, traceability, usability, robustness, and explainability across the AI lifecycle [66].

### 9.8. Regulatory Classification and Accountability

An AI system that recommends or ranks patient-specific AAD therapy may meet the definition of medical device software and will require regulation according to its intended purpose and clinical risk. In the European Union, AI systems incorporated into medical devices requiring third-party conformity assessment may also be classified as high risk under the AI Act, with requirements concerning risk management, documentation, human oversight, accuracy, robustness, cybersecurity, logging, and post-market monitoring [67,68]. However, the relationship between the AI Act and sector-specific medical device legislation continues to evolve as the relevant European Union instruments are amended. Therefore, the applicable regulatory obligations should be determined from the latest official consolidated text on EUR-Lex, rather than by relying on the AI Act Explorer alone [69]. In the United States, the Food and Drug Administration has issued final guidance on predetermined change-control plans for AI-enabled devices and draft lifecycle guidance addressing development, validation, and continuing risk management [67]. Responsibility must nevertheless remain traceable across developers, manufacturers, healthcare institutions, deployers, and clinicians. Adaptive updating should be auditable, version-controlled, and restricted to prospectively specified and validated modifications.

### 9.9. Clinical Utility and Cost-Effectiveness

Regulatory authorization and statistical performance do not demonstrate clinical value. AI-supported prescribing should improve patient-important outcomes, such as symptom control, AF burden, quality of life, serious toxicity, hospitalization, and treatment persistence, without creating excessive testing, alerts, inequity, or clinician workload. Evaluation should include workflow impact, automation bias, downstream resource use, opportunity costs, and cost-effectiveness compared with standard care, which can be assessed only once a clinical effect has been demonstrated. Contemporary evaluation frameworks recommend prospective monitoring throughout deployment and assessment of health, cost, and resource consequences rather than reliance on model accuracy alone [70,71]. Comparative clinical-impact trials provide the strongest evidence on whether AI makes antiarrhythmic therapy safer, more effective, and more efficient. Carefully emulated target trials and prospective registry-based evaluation can provide complementary evidence, particularly for rare outcomes and long-term safety (Table 2).

**Table 2:** Principal challenges, potential consequences, and required safeguards for artificial intelligence (AI)-supported antiarrhythmic drug therapy.

Challenge	Mechanism of Failure	Potential Clinical Consequence	Required Methodological or Governance Safeguards	Relevant Frameworks or Guidance
**Limited and fragmented evidence**	Models are frequently developed retrospectively in small, single-center, or highly selected populations. Evidence may be transferred from AF detection, ablation, general pharmacovigilance, or drug-induced QT studies without direct validation for AAD selection	Premature claims of clinical readiness; use of models outside their intended purpose; recommendations based on indirect or weak evidence	Clearly define the intended use; distinguish AF-specific evidence from transferable evidence; prioritize multicenter development, external validation, prospective evaluation, and randomized clinical-impact studies	DECIDE-AI; CONSORT-AI; TRIPOD+AI [40,62]
**Confounding by indication**	AAD allocation in routine care reflects structural disease, ventricular function, previous treatment failure, clinician preference, drug availability, access to ablation, and patient characteristics	Spurious associations between a drug and poor outcomes; incorrect ranking of treatments; reinforcement of historical prescribing patterns	Emulate a clearly specified target trial; define eligibility, treatment assignment, time zero, follow-up, and outcomes; use appropriate propensity, weighting, marginal structural, or causal machine learning methods; perform sensitivity analyses for unmeasured confounding	Target trial framework [63]
**Incomplete or inaccurate treatment exposure data**	Prescriptions may not reflect dispensing, ingestion, dose changes, treatment interruptions, cumulative exposure, or adherence. Drugs and clinical events outside the primary health system may be missed	Misclassification of response or toxicity; inaccurate dose–response relationships; false attribution of treatment failure	Integrate prescribing, dispensing, medication-reconciliation, drug-concentration, device, and patient-reported data; model adherence and exposure as time-varying variables; verify treatment discontinuation and reasons for change	TRIPOD+AI; FUTURE-AI [40,66]
**Inconsistent definitions of treatment success**	Studies use different outcomes, including acute cardioversion, time to first recurrence, AF burden, symptom improvement, hospitalization, treatment discontinuation, and adverse events	Models may appear discordant despite addressing different clinical questions; pooled training data may combine non-equivalent outcomes; recommendations may not align with patient goals	Predefine treatment-specific and patient-important outcomes; standardize recurrence and AF burden definitions; report monitoring technology and analyzable observation time; distinguish rhythm efficacy, symptoms, safety, and treatment persistence	AF burden consensus recommendations [64]; TRIPOD+AI [40]
**Dynamic and competing outcomes**	AF recurrence, adverse effects, adherence, organ dysfunction, drug switching, cardioversion, ablation, hospitalization, substrate progression, and death interact over time	Biased prediction from static baseline models; failure to recognize changing benefit-risk balance; misleading estimates when competing events prevent outcome observation	Use longitudinal, multistate, joint, competing risk, and dynamic prediction models; record time-stamped treatment and monitoring data; update predictions after clinically important changes	TRIPOD+AI; FUTURE-AI [40,66]
**Dataset shift and limited generalizability**	Patient populations, ECG systems, laboratory platforms, monitoring devices, clinical workflows, prescribing practices, and drug availability differ across institutions and countries and may change over time	Loss of discrimination or calibration; unsafe recommendations in new settings; progressive deterioration after deployment	Perform geographic, temporal, and technological external validation; assess calibration and subgroup performance locally; monitor drift; define conditions requiring recalibration, model updating, or abstention	TRIPOD+AI; PROBAST+AI; FUTURE-AI [40,65,66]
**Bias, inequity, and under-representation**	Women, older adults, ethnic minorities, patients with multimorbidity, advanced kidney disease, or limited digital access may be under-represented or have systematically poorer quality data	Differential error rates; underestimation of toxicity; inappropriate treatment denial or selection; widening of disparities through dependence on wearables or advanced imaging	Ensure representative recruitment and outcome ascertainment; evaluate intersectional subgroup performance and calibration; use clinically justified thresholds; provide non-digital alternatives; monitor access and downstream harms	FUTURE-AI; Equity in Medical Devices review [66,72]
**Missing, noisy, or selectively collected data**	Testing and monitoring intensity are influenced by disease severity, treatment choice, access, and clinician concern. Wearable data may be incomplete or artefactual.	Models may interpret absence of measurement as absence of risk; intensively monitored patients may appear to have more adverse events; recommendations may favor patients with richer data	Represent missingness explicitly; distinguish unavailable, unmeasured, and normal values; assess informative monitoring; use multimodal quality control procedures; require model abstention when essential inputs are unreliable	TRIPOD+AI; FUTURE-AI [40,66]
**Poor calibration and unquantified uncertainty**	A model may rank patients correctly while systematically overestimating or underestimating absolute efficacy or toxicity. Predictions may be extrapolated beyond the development population	Incorrect benefit–risk comparisons; inappropriate drug selection or monitoring intensity; false confidence in individual recommendations	Assess calibration in the large, calibration slope, and clinically relevant probability ranges; provide confidence or prediction intervals where appropriate; detect out-of-distribution cases; implement abstention and clinician review thresholds	TRIPOD+AI; PROBAST+AI; FUTURE-AI [40,65,66]
**Limited explainability and automation bias**	Opaque recommendations may not reveal whether predictions are driven by clinically plausible variables, data artefacts, or hidden institutional signals. Clinicians may accept algorithmic rankings without adequate scrutiny.	Failure to detect model errors; inappropriate treatment despite contraindications or unrepresented circumstances; erosion of clinician responsibility	Provide drug-specific contributing factors, alternatives, uncertainty, and reasons for abstention; design interfaces that support verification; evaluate clinician interaction, overrides, and error recognition; train users in model limitations	DECIDE-AI; FUTURE-AI [61,66]
**Regulatory classification and model updating**	Patient-specific treatment ranking may constitute medical-device software. Adaptive algorithms may change performance after authorization.	Deployment without appropriate conformity assessment; uncontrolled performance changes; unclear versioning and accountability	Define intended purpose and risk classification; maintain technical documentation, validation evidence, change control procedures, version control, audit logs, human oversight, and post-market monitoring; prospectively specify permitted updates	EU AI Act; MDR/IVDR–AI Act guidance; FDA predetermined change-control and lifecycle guidance [66,67,73,74]
**Cybersecurity, privacy, and data governance**	Multimodal systems may require linkage of clinical, ECG, imaging, wearable, genomic, and longitudinal data across institutions and devices.	Unauthorized access, data leakage, manipulation of model inputs, service disruption, re-identification, and loss of patient trust	Apply data minimization, secure architecture, access control, encryption, provenance tracking, adversarial testing, breach response plans, and compliance with applicable data protection legislation	EU AI Act; FUTURE-AI; relevant data-protection and medical-device cybersecurity requirements [66,74]
**Unclear accountability for recommendations and harm**	Responsibility may be distributed among software developers, manufacturers, institutions, clinicians, and data providers.	Delayed recognition of failure; uncertainty regarding liability; inappropriate delegation of clinical judgment to the system	Define responsibilities contractually and operationally; retain clinician authority for final decisions; record recommendations, inputs, explanations, overrides, and outcomes; establish incident-reporting and escalation procedures	EU AI Act; FDA lifecycle guidance; FUTURE-AI [66,73,74]
**Alert fatigue and poor workflow integration**	Excessive warnings, duplicated interaction alerts, poorly timed recommendations, or separate interfaces increase cognitive and administrative burden	Alert overrides, reduced trust, missed clinically important warnings, workflow disruption, and reduced adoption	Integrate decision support into existing workflows; prioritize alerts according to severity and actionability; minimize duplicate data entry; conduct usability and human factors testing; monitor override patterns	DECIDE-AI; AHA pragmatic evaluation guidance [70]
**Uncertain clinical utility and cost-effectiveness**	Improved discrimination may not change treatment, outcomes, resource use, or patient experience. Advanced testing and monitoring may increase costs without proportional benefit.	Technological adoption without meaningful clinical benefit; unnecessary testing; increased inequalities and opportunity costs	Conduct prospective comparative impact trials; evaluate patient-important outcomes, workflow, clinician workload, downstream procedures, resource use, and cost-effectiveness; compare with optimized conventional care	CONSORT-AI; NICE Evidence Standards Framework; AHA pragmatic evaluation guidance [70,71]

The challenges listed in this table are interdependent. For example, under-representation can contribute to dataset shift, poor calibration, and inequitable access, while incomplete exposure data can amplify confounding by indication and distort estimates of efficacy and toxicity. The table applies specifically to AI systems intended to support selection, dosing, initiation, monitoring, or modification of antiarrhythmic drug (AAD) therapy in atrial fibrillation (AF). It does not imply that all systems require the same regulatory pathway; classification depends on the intended purpose, level of autonomy, clinical context, and associated risk. “External validation” should involve evaluating a locked model on data that are meaningfully independent of the development dataset. Random division of records from the same institution or data source does not adequately test geographic, temporal, technological, or healthcare-system transportability. “Calibration” refers to agreement between predicted probabilities and observed event frequencies. A model may demonstrate acceptable discrimination while remaining unsuitable for clinical decision-making because its absolute risk estimates are inaccurate. “Out-of-distribution detection” refers to recognition that a patient, drug, dose, healthcare setting, data modality, or clinical circumstance differs materially from those represented during model development. In these circumstances, the system should abstain, downgrade confidence, or require explicit specialist review. “Target trial emulation” requires explicit specification of eligibility criteria, treatment strategies, treatment assignment, time zero, follow-up, outcomes, causal contrast, and analytical approach before observational data are analyzed. Patient-important outcomes should include not only rhythm efficacy but also AF burden, symptoms, functional status, quality of life, serious cardiac and extracardiac adverse effects, hospitalization, treatment discontinuation, need for ablation, healthcare utilization, and treatment burden. AHA—American Heart Association; EU—European Union; FDA—United States Food and Drug Administration; IVDR—In Vitro Diagnostic Medical Devices Regulation; MDR—Medical Devices Regulation.

## 10. Research Agenda for Artificial Intelligence-Guided Antiarrhythmic Therapy

### 10.1. Minimum Requirements for Future Datasets

Progress will require purpose-built, multicenter datasets rather than opportunistic extraction of fragmented routine care records. Cohorts should represent different healthcare systems, prescribing cultures, demographic groups, comorbidity profiles, and levels of access to catheter ablation and continuous monitoring. Drug exposure must be recorded longitudinally, including indication, starting dose, titration, treatment interruptions, cumulative exposure, dispensing, adherence, drug concentrations where available, and reasons for discontinuation. Serial 12-lead and ambulatory ECG should be linked with renal and hepatic function, electrolytes, concomitant medications, symptoms, AF burden, cardioversions, hospitalizations, ablation, and cardiac and extracardiac adverse events. Harmonized data definitions, prespecified analysis plans, transparent reporting, and formal risk of bias assessment should follow TRIPOD+AI and PROBAST+AI principles [40,65].

Meeting these requirements will nevertheless be difficult while relevant longitudinal data remain fragmented across institutions and regulatory jurisdictions. Privacy-preserving multicenter learning, particularly federated learning, could provide a practical infrastructure for developing clinically credible AAD models without routinely transferring patient-level data to a centralized database. Each center would retain its own data locally and share only model parameters or training updates. This could support developing drug-specific models to predict both efficacy and toxicity while reducing the need to centralize highly sensitive multimodal records. However, federated learning should not be viewed as an automatic solution to either privacy or generalizability. Participating centers may differ substantially in patient profiles, drug access, outcome definitions, ECG equipment, follow-up intensity, and local treatment practices [75]. These differences can lead to heterogeneous datasets and models that perform inconsistently across sites. For this reason, federated AAD models should be evaluated on completely independent centers, with site-level calibration, subgroup analyses, secure aggregation, and formal testing against privacy attacks. Their performance should also be monitored prospectively, particularly for drifting over time. At present, most cardiovascular AI evidence remains retrospective and lacks the level of validation needed for routine implementation. Federated learning is therefore a promising research infrastructure, but not, by itself, proof that a model will transfer safely and reliably into clinical practice [76].

### 10.2. Development of Comparative Treatment Models

Future models should not merely predict recurrence among patients receiving one observed drug. They should estimate outcomes for the same patient under several eligible AADs, doses, and non-pharmacological alternatives. Separate predictions are required for rhythm efficacy, AF burden reduction, proarrhythmia, organ toxicity, treatment discontinuation, and monitoring burden. Randomized trial data should be prioritized, while observational analyses should emulate explicitly defined target trials. Treatment exposure required particular care: time zero should be defined as the point at which eligibility, exposure, and follow-up are assessed simultaneously rather than selected retrospectively; exposure windows should be prespecified; discontinuation should be recorded with its reason, and for the drugs with tissue accumulation, cumulative exposure rather than current dose is the physiologically relevant quantity [63]. Causal ML methods may help characterize heterogeneous treatment effects, but individualized estimates must include uncertainty and should not be interpreted as causal truths when relevant confounders are unavailable [77].

### 10.3. Prospective Validation

Before influencing care, a locked model should undergo silent prospective evaluation within the intended workflow. Predictions should be generated without being shown to clinicians and compared with observed efficacy, toxicity, and treatment changes. This phase should assess data availability, calibration, subgroup performance, dataset shift, latency, missingness, and how often the model encounters unsupported patients. Subsequent clinician-facing studies should examine usability, automation bias, overrides, model abstention, and unintended workflow effects in accordance with DECIDE-AI [61].

### 10.4. Artificial Intelligence-Guided Clinical Trials

Translation should proceed through six stages:

1. Retrospective model development with internal validation;
2. Geographic and temporal external validation;
3. Silent prospective observational validation;
4. Early clinical impact and human factors evaluation;
5. A randomized trial comparing AI-assisted with guideline-based conventional prescribing;
6. Post-deployment surveillance for performance drift, inequity, safety events, and changing clinical practice.

**Figure 7:**
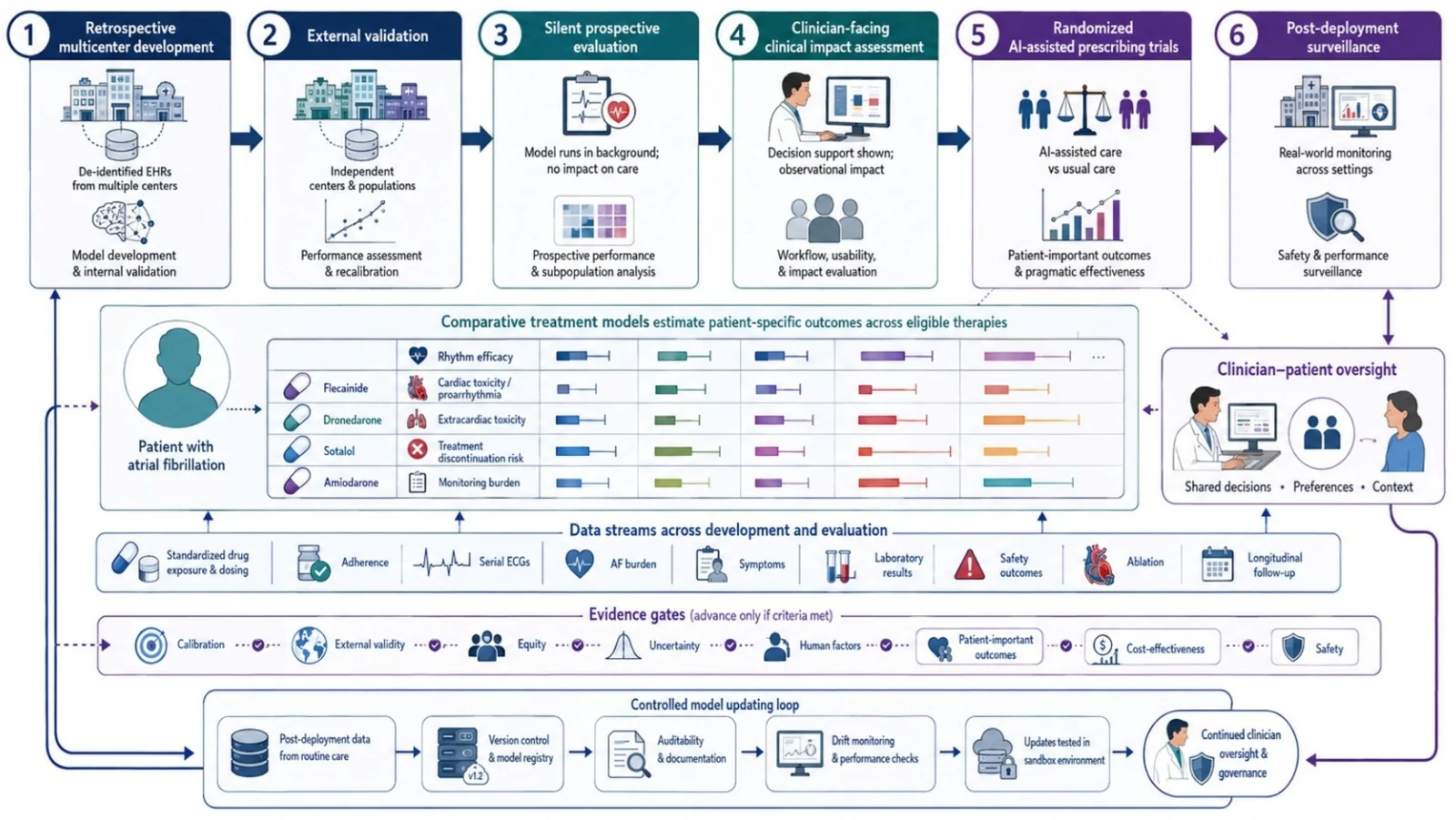
Translational roadmap for artificial intelligence (AI)-guided antiarrhythmic drug (AAD) therapy in atrial fibrillation (AF). The figure depicts a six-stage pathway progressing from retrospective multicenter development through external validation, silent prospective evaluation, clinician-facing clinical impact assessment, randomized AI-assisted prescribing trials, and post-deployment surveillance. A parallel data stream includes standardized drug exposure and dosing, adherence, serial ECGs, AF burden, symptoms, laboratory results, safety outcomes, ablation, and longitudinal follow-up. Comparative treatment models estimate patient-specific efficacy, cardiac and extracardiac toxicity, treatment discontinuation, and monitoring burden across multiple eligible drugs. Advancement between stages requires predefined evidence gates covering calibration, external validity, equity, uncertainty, human factors performance, patient-important outcomes, cost-effectiveness, and safety. A final feedback loop shows controlled model updating using post-deployment data, with version control, auditability, drift monitoring, and continued clinician oversight. ECG—electrocardiogram; EHR—electronic health record.

This staged translational pathway, together with the evidence gates and post-deployment model update loop, is summarized in Figure 7.

Randomized protocols and reports should specify the AI intervention, required inputs, workflow integration, human-AI interaction, error handling, and update procedures according to SPIRIT-AI and CONSORT-AI [54,78].

### 10.5. Standardized Core Outcomes

Trials should simultaneously evaluate maintenance of sinus rhythm, AF burden, longest AF episode, symptoms, functional status, quality of life, cardioversion, hospitalization, proarrhythmia, extracardiac toxicity, treatment discontinuation, and need for ablation. AF burden should be reported as the proportion of monitored time spent in AF during a specified observation period, together with monitoring duration and the longest episode, thereby reducing dependence on time to first recurrence alone [62].

### 10.6. Toward a Continuously Learning Therapeutic System

The long-term objective is a bounded, continuously evaluated system that updates comparative treatment estimates when new rhythm, physiological, laboratory, adherence, and adverse event data become available. Learning should not mean uncontrolled autonomous modification. Model versions, permitted updates, calibration, drift, failure cases, and clinical outcomes must remain traceable throughout the system lifecycle, consistent with trustworthy AI and regulatory lifecycle principles [66,73]. The decisive advance will occur when AI no longer predicts outcomes only once, but supports repeated, transparent reassessment of the safest and most effective rhythm control strategy as both the patient and AF substrate evolve.

## 11. Conclusions

Antiarrhythmic drug therapy remains an important component of contemporary AF management, including pharmacological cardioversion, maintenance of sinus rhythm, treatment surrounding catheter ablation, and rhythm control in patients who cannot or do not wish to undergo an invasive procedure. Nevertheless, drug selection remains incompletely personalized. Current algorithms primarily identify agents that can be used safely based on structural heart disease, ventricular function, coronary disease, organ function, conduction abnormalities, and established contraindications, but offer limited guidance regarding which eligible drug offers the greatest individual net benefit. AI may enable a transition from safety-based eligibility toward patient-specific estimation of antiarrhythmic efficacy, AF-burden reduction, proarrhythmia, extracardiac toxicity, treatment discontinuation, and monitoring requirements. Potential applications extend from initial patient and drug selection to dose optimization, risk-adapted initiation, continuous rhythm and safety surveillance, and longitudinal treatment modification. Computational electrophysiology and digital twins may complement data-driven models by allowing virtual comparison of drug effects within patient-specific atrial substrates. Existing studies have demonstrated proof of concept for predicting drug-induced QT prolongation, amiodarone-associated thyroid dysfunction, and simulated amiodarone response. However, these advances do not yet justify autonomous or routine AI-guided prescribing. Available evidence remains predominantly retrospective, application-specific, and non-comparative. Clinical adoption will require multicenter datasets, external and silent prospective validation, individualized treatment-effect estimation, transparent uncertainty reporting, randomized clinical impact trials, and post-deployment surveillance. AI should ultimately support, not replace, shared clinician-patient decisions as therapeutic goals, AF substrate, organ function, treatment response, and patient preferences evolve over time.

## Figures and Tables

**Table 1:** Data modalities and artificial intelligence approaches for precision antiarrhythmic therapy in atrial fibrillation.

Data Modality	Representative Inputs	Suitable AI and Analytical Approaches	Potential Applications to Antiarrhythmic Therapy	Principal Sources of Bias, Error, or Uncertainty	Evidence Level and Type
**Clinical and electronic health record data**	Age, sex, AF type and duration, cardiovascular diagnoses, ventricular function, renal and hepatic indices, electrolytes, medication exposure, drug dose, comorbidities, previous cardioversions, hospitalizations, prior AAD response, treatment discontinuation, and documented adverse events	Gradient boosting, random forests, regularized regression, neural networks, survival analysis	Screening for drug eligibility and contraindications; prediction of QT prolongation, bradyarrhythmia, organ toxicity, treatment discontinuation, and hospitalization; identification of dose constraints and drug–drug or drug–disease interactions; estimation of comparative treatment effects	Missing or inaccurately coded diagnoses; incomplete medication and adherence data; variation in laboratory timing; confounding by indication; clinician- and institution-specific prescribing patterns; informative loss to follow-up; limited capture of events occurring outside the healthcare system	Predictive safety: Early clinical evidence. Feasibility has been demonstrated for drug-induced QT risk prediction, but direct comparison and selection among alternative AADs remain largely undeveloped [31]
**Standard 12-lead ECG**	Rhythm, P-wave characteristics, PR interval, QRS duration and morphology, QT/QTc interval, T-wave morphology, ventricular rate, ectopy, conduction abnormalities, repolarization heterogeneity, and serial changes after drug administration	Conventional feature-based models, convolutional neural networks, and foundation models	Baseline ventricular safety assessment; prediction of drug-induced QT prolongation or QRS widening; identification of latent repolarization vulnerability; recognition of pharmacodynamic response following initiation; support for dose adjustment or treatment discontinuation	Device and lead-placement variability; heart-rate dependence of intervals; limitations of automated QT correction; rhythm-dependent measurement error; hidden demographic or institutional signals; limited representation of rare proarrhythmic outcomes	Predictive safety: Moderate proof-of-concept evidence for safety applications. Deep learning ECG models can detect drug-related repolarization patterns, but prospective evidence for guiding AAD selection is lacking [32,38]
**Ambulatory, high-resolution, and signal-averaged ECG**	AF episode onset and termination, AF burden, ventricular rate behavior, pauses, ectopy, rate-dependent QRS widening, dynamic QT changes, beat-to-beat repolarization variability, late potentials, and lower amplitude atrial activity	Time series models, recurrent neural networks, temporal convolutional networks	Detection of early efficacy or non-response; identification of rate-dependent conduction slowing or repolarization instability; prediction of recurrent AF; assessment of within-patient changes following dose escalation; surveillance for intermittent bradyarrhythmia or proarrhythmia	Noise, artefact, incomplete wear time, uncertain episode annotation, variation in sampling frequency, device-specific algorithms, missing contextual information, and low event rates for serious ventricular arrhythmias	Predictive safety: Emerging clinical evidence. Continuous QT assessment after class III AAD initiation is feasible, but its effect on clinical outcomes has not yet been established [33]
**Wearable and implantable sensor data**	Photoplethysmography, single-lead ECG, AF burden, episode duration, ventricular rate patterns, resting heart rate, physical activity, sleep duration and regularity, circadian recurrence, autonomic surrogates, symptom annotations, and device use patterns	Time-series analysis, multimodal deep learning, digital phenotyping	Quantification of treatment-related reduction in AF burden; recognition of circadian or trigger-related recurrence; symptom-rhythm correlation; early detection of loss of efficacy; monitoring of treatment tolerance; indirect recognition of adherence-related patterns; dynamic reassessment after dose changes	Motion artefact, missing wear time, variable sensor accuracy, false-positive AF detection, unequal access and digital literacy, algorithm changes, device replacement, selective use during symptoms, and uncertain interpretation of autonomic or adherence surrogates	Prognostic: Early-to-moderate evidence for rhythm monitoring, but limited evidence for drug optimization. AF-burden estimation is feasible, although direct use for individualized AAD adjustment remains investigational [34]
**Echocardiography and cardiac computed tomography**	Left atrial dimensions and volume, atrial strain and mechanical function, left ventricular ejection fraction, valvular disease, filling pressures, atrial anatomy, pulmonary vein anatomy, and epicardial adipose tissue	Radiomics, automated segmentation, deep learning image analysis	Assessment of drug eligibility; estimation of atrial remodeling and likelihood of durable rhythm control; differentiation of trigger-dominant from substrate-dominant AF; identification of patients in whom ablation or substrate modification may be preferable to repeated drug escalation	Operator and acquisition variability; vendor dependence; inconsistent chamber and strain measurements; limited temporal sampling; contrast and radiation constraints for computed tomography; confounding by loading conditions	Prognostic: Established clinical relevance of imaging variables but limited direct AI evidence for AAD selection. Most applications remain prognostic or supportive rather than treatment comparative
**Cardiac magnetic resonance and electro-anatomical mapping**	Atrial fibrosis, scar distribution, chamber geometry, wall thickness, bipolar and unipolar voltage, activation times, conduction velocity, electrogram fractionation, rotational activity, and low voltage areas	Deep learning segmentation, graph neural networks, spatial clustering, image-map registration	Characterization of atrial substrate; prediction of conversion and maintenance efficacy; mechanistic comparison of alternative drugs; construction of patient-specific virtual atria; simulation of drug effects on conduction, refractoriness, and AF sustainability	Limited availability; invasive acquisition for mapping; variation in voltage thresholds and mapping rhythm; registration error; incomplete biatrial coverage; imaging artefact; computational assumptions; uncertain parameter identifiability	Prognostic: Proof-of-concept evidence. Patient-specific digital twins have simulated amiodarone response, but available studies are retrospective, small, and not yet suitable for routine prescribing [7,35]
**Molecular, genomic, pharmacogenomic, and other omics data**	Ion-channel variants, *PITX2*-related variants, genes affecting calcium handling or repolarization, CYP and transporter polymorphisms, polygenic scores, circulating proteins, metabolites, inflammatory markers, transcriptomic profiles, and drug concentrations	Penalized regression, polygenic modeling, multi-omics integration, and multimodal deep learning	Identification of altered drug sensitivity or metabolism; prediction of exposure, efficacy, and proarrhythmia; refinement of atrial mechanistic phenotype; detection of biological pathways associated with non-response or toxicity	Small sample sizes; multiple testing; ancestry-dependent effects; batch and platform variability; uncertain functional interpretation; tissue-blood discordance; low prevalence of actionable variants; weak replication across cohorts	Prognostic: Exploratory evidence. Associations between selected variants and AAD response have been reported, but no genomic or omics model currently supports routine AF drug selection [27]
**Integrated multimodal data**	Combined clinical, longitudinal, ECG, wearable, imaging, mapping, genomic, laboratory, and treatment exposure data	Multimodal transformers, cross-modal representation learning, foundation models	Comparative estimation of efficacy and toxicity across eligible AADs; individualized dose and initiation planning; integration of atrial benefit with ventricular and extracardiac safety; dynamic treatment ranking; identification of patients better suited to ablation; continuous updating of recommendations	Data synchronization problems; modality-specific missingness; overfitting; poor interpretability; dataset shift; limited sample size relative to model complexity; unequal availability of advanced tests; propagation of bias from individual data sources	Comparative: Conceptually strongest but clinically least validated approach. Multimodal and foundation models are advancing rapidly, yet prospective AF-specific treatment selection studies remain absent or exceptionally limited [37,38]
**Post-treatment longitudinal feedback data**	Serial ECGs, AF burden, symptoms, quality of life, laboratory surveillance, organ-function changes, adverse events, adherence, dose modifications, cardioversions, hospitalizations, ablation, and reasons for treatment discontinuation	Dynamic prediction, online learning, joint longitudinal time-to-event modeling	Early identification of non-response or emerging toxicity; adjustment of dose and monitoring frequency; recognition of changing benefit-risk balance; decision to switch drugs, discontinue treatment, or proceed to ablation; continuous updating of patient-specific models	Irregular follow-up; treatment changes driven by evolving clinical status; informative missingness; delayed documentation; reward misspecification in reinforcement learning; risk of unsafe recommendations when models extrapolate beyond observed care	Comparative: Largely conceptual for adaptive prescribing. Longitudinal monitoring is clinically established, but AI-driven treatment adaptation requires prospective validation and controlled impact evaluation

The table summarizes potential data and analytical approaches specifically for artificial intelligence (AI)-supported antiarrhythmic drug (AAD) therapy in atrial fibrillation (AF). “Potential therapeutic applications” include both currently investigated uses and plausible future applications. “Current evidence level” was assessed qualitatively according to the proximity of available evidence to actual antiarrhythmic prescribing: exploratory—biological association or retrospective feature discovery without a validated therapeutic model; proof of concept—demonstration that the method can predict or simulate a relevant response in a limited retrospective or experimental setting; early clinical evidence—evaluation using clinical data with internal or limited external validation, but no demonstrated effect on prescribing or patient outcomes; moderate evidence—reproducible clinical prediction across relevant cohorts or settings, without prospective proof that model-guided treatment improves outcomes; established clinical application: prospective evidence of clinical utility and incorporation into routine care. No AI application identified in this review currently reaches the final category for comparative selection among AADs in AF. CYP—cytochrome P450; QTc—heart-rate-corrected QT interval.

## Data Availability

No new data were created or analyzed in this study. Data sharing is not applicable to this article.

## Supplementary Materials

The following supporting information can be downloaded at https://www.mdpi.com/article/10.3390/medicina62091783/s1: Supplementary Table S1. Database-specific literature-search strategies for artificial intelligence (AI) and computational modeling in antiarrhythmic drug (AAD) therapy for atrial fibrillation (AF).

## Abbreviations

The following abbreviations are used in this manuscript:

AF	Atrial Fibrillation
EAST-AFNET 4	The Early Treatment of Atrial Fibrillation for Stroke Prevention Trial
AADs	antiarrhythmic drugs
AI	Artificial intelligence
IEEE	Institute of Electrical and Electronics Engineers
CYP	CYP—cytochrome P450
QTc	heart-rate-corrected QT interval
AHA	American Heart Association
EU	European Union
FDA	United States Food and Drug Administration
IVDR	In Vitro Diagnostic Medical Devices Regulation
MDR	Medical Devices Regulation
CT	Computed tomography
DCCV	Direct current cardioversion
MRI	Magnetic resonance imaging
ED	Emergency Department
LVEF	Left ventricular ejection fraction
PD	Pharmacodynamic
RNA	Ribonucleic acid
SR	Sinus rhythm
TdP	Torsades de pointes
DTI	Diffusion tensor imaging
dT	delta time
dV	Delta amplitude
EAM	electro-anatomical mapping
EP	electrophysiological
I_CaL_	L-type calcium current
I_Kr_	Rapid component of the delayed rectifying potassium current
I_Ks_	Slow component of the delayed rectifying potassium current
I_Kur_	Ultrarapid component of the delayed rectifying potassium current
I_Na_	voltage-gated sodium current
I_to_	Transient outward potassium current
LGE	Late gadolinium enhancement
EHR	Electronic health record
ECG	Electrocardiogram
