# Supplementary material for: Artificial Intelligence for Precision Antiarrhythmic Drug Therapy in Atrial Fibrillation: From Recurrence Prediction to Comparative Treatment Selection
Source: Medicina (Kaunas). 2026 Sep 16;62(9):1783. doi: 10.3390/medicina62091783 (PMC13609021; doi:10.3390/medicina62091783)
Supplement: Supplementary file 1 [file medicina-62-01783-s001.zip › medicina-4549647-supplementary.pdf]

SUPPLEMENTARY MATERIAL

Supplementary Table S1. Database-specific literature-search strategies for artificial intelligence (AI) and computational modeling in antiarrhythmic drug (AAD) therapy for atrial fibrillation (AF).

Database and platform	Search fields and coverage	Complete search strategy	Restrictions
**PubMed/MEDLINE**	Medical Subject Headings and title/abstract fields; database inception to final search date	**Concept 1 – Atrial fibrillation:** ("Atrial Fibrillation"[Mesh] OR "atrial fibrillation" OR "atrial fibrillations") **AND Concept 2 – Antiarrhythmic therapy:** ("Anti-Arrhythmia Agents"[Mesh] OR antiarrhythmic* OR anti-arrhythmic* OR "rhythm control" OR flecainide OR propafenone OR sotalol OR dofetilide OR dronedarone OR amiodarone OR disopyramide OR quinidine OR ibutilide OR vernakalant) **AND Concept 3 – AI, prediction, and computational modeling:** ("Artificial Intelligence"[Mesh] OR "Machine Learning"[Mesh] OR "Deep Learning"[Mesh] OR "artificial intelligence" OR "machine learning" OR "deep learning" OR neural network* OR algorithm* OR "predictive model*" OR "risk prediction" OR "treatment response" OR "treatment response prediction" OR "drug response" OR "drug selection" OR "individualized treatment effect*" OR "precision pharmacotherapy" OR "precision medicine" OR pharmacovigilance OR proarrhythm* OR "drug-induced arrhythmia*" OR "QT prolongation" OR "toxicity prediction" OR "adverse event prediction" OR "computational electrophysiology" OR "computational model*" OR "mechanistic model*" OR "in silico" OR "virtual patient*" OR "digital twin*")	No language, publication-status, study design, or human-only restrictions

Database and platform	Search fields and coverage	Complete search strategy	Restrictions
**Scopus**	Article title, abstract, and author keywords; database inception to final search date	TITLE-ABS-KEY("atrial fibrillation") AND TITLE-ABS-KEY(antiarrhythmic* OR "anti-arrhythmic" OR "rhythm control" OR flecainide OR propafenone OR sotalol OR dofetilide OR dronedarone OR amiodarone OR disopyramide OR quinidine OR ibutilide OR vernakalant) AND TITLE-ABS-KEY("artificial intelligence" OR "machine learning" OR "deep learning" OR "neural network*" OR algorithm* OR "predictive model*" OR "risk prediction" OR "treatment response" OR "treatment response prediction" OR "drug response" OR "drug selection" OR "individualized treatment effect*" OR "precision pharmacotherapy" OR "precision medicine" OR pharmacovigilance OR proarrhythm* OR "drug-induced arrhythmia*" OR "QT prolongation" OR "toxicity prediction" OR "adverse event prediction" OR "computational electrophysiology" OR "computational model*" OR "mechanistic model*" OR "in silico" OR "virtual patient*" OR "digital twin*")	No language, document type, source type, or subject area restrictions
**Web of Science Core Collection**	Topic field, comprising title, abstract, author keywords, and Keywords Plus; database inception to final search date	TS=("atrial fibrillation") AND TS=(antiarrhythmic* OR "anti-arrhythmic" OR "rhythm control" OR flecainide OR propafenone OR sotalol OR dofetilide OR dronedarone OR amiodarone OR disopyramide OR quinidine OR ibutilide OR vernakalant) AND TS=("artificial intelligence" OR "machine learning" OR "deep learning" OR "neural network*" OR algorithm* OR "predictive model*" OR "risk prediction" OR "treatment response" OR "treatment response prediction" OR "drug response" OR "drug selection" OR "individualized treatment effect*" OR "precision pharmacotherapy" OR "precision medicine" OR pharmacovigilance OR proarrhythm* OR "drug-induced arrhythmia*" OR "QT prolongation" OR "toxicity prediction" OR "adverse event prediction" OR "computational	All Web of Science Core Collection indexes; no language or document type restrictions

Database and platform	Search fields and coverage	Complete search strategy	Restrictions
		electrophysiology" OR "computational model*" OR "mechanistic model*" OR "in silico" OR "virtual patient*" OR "digital twin*")	
**IEEE Xplore Digital Library**	Command Search across publication metadata; database inception to final search date	("atrial fibrillation") AND (antiarrhythmic* OR "anti-arrhythmic" OR "rhythm control" OR flecainide OR propafenone OR sotalol OR dofetilide OR dronedarone OR amiodarone OR disopyramide OR quinidine OR ibutilide OR vernakalant) AND ("artificial intelligence" OR "machine learning" OR "deep learning" OR "neural network*" OR algorithm* OR "predictive model*" OR "risk prediction" OR "treatment response" OR "drug response" OR "drug selection" OR "precision pharmacotherapy" OR pharmacovigilance OR proarrhythm* OR "drug-induced arrhythmia*" OR "QT prolongation" OR "computational electrophysiology" OR "computational model*" OR "mechanistic model*" OR "in silico" OR "virtual patient*" OR "digital twin*")	Metadata search; all IEEE content types retained, including journal articles and conference proceedings; no language restrictions

Supplementary targeted-search modules

Because potentially relevant evidence may not simultaneously mention AF, an individual antiarrhythmic drug, and an AI-related term, the principal database search was supplemented by the following targeted searches.

Search module	Purpose	Search concepts
**A. AI and antiarrhythmic drug safety beyond AF-specific populations**	To identify transferable models for drug-induced QT prolongation, ventricular proarrhythmia, bradyarrhythmia, organ toxicity, and pharmacovigilance	(antiarrhythmic* OR "drug-induced QT prolongation" OR "long QT" OR proarrhythm* OR torsades OR "drug-induced arrhythmia*" OR "drug toxicity" OR pharmacovigilance) AND ("artificial intelligence" OR "machine learning" OR "deep learning" OR "neural network*" OR "predictive model*" OR algorithm*)
**B. Computational electrophysiology and digital twins**	To identify mechanistic and patient-specific simulations of drug response that may not be indexed as AI	("atrial fibrillation" OR atrial) AND (antiarrhythmic* OR "drug effect*" OR flecainide OR propafenone OR sotalol OR dofetilide OR dronedarone OR amiodarone) AND ("computational electrophysiology" OR "computational model*" OR "mechanistic model*" OR "in silico" OR "virtual patient*" OR "digital twin*" OR "population of models")
**C. Precision treatment-response estimation**	To identify studies estimating differential or individualized response to alternative therapies	("atrial fibrillation") AND (antiarrhythmic* OR "rhythm control") AND ("treatment response" OR "drug response" OR "drug selection" OR "comparative effectiveness" OR "heterogeneous treatment effect*" OR "individualized treatment effect*" OR "causal machine learning" OR "reinforcement learning" OR "precision pharmacotherapy")
**D. Methodological and implementation literature**	To identify reporting standards, validation frameworks, clinical decision support methods, and regulatory considerations relevant to AI-supported prescribing	("artificial intelligence" OR "machine learning") AND ("clinical decision support" OR "treatment recommendation" OR "clinical prediction model" OR calibration OR explainability OR uncertainty OR "external validation" OR implementation OR regulation OR "software as a medical device")

Additional evidence identification procedures

Backward reference screening was performed for all included primary studies and major reviews. Forward citation tracking was conducted in Scopus, Web of Science, or Google Scholar, as appropriate. Targeted searches were also undertaken for contemporary AF guidelines, antiarrhythmic drug consensus statements, reporting and validation frameworks for clinical AI, and regulatory documents from relevant professional societies and regulatory authorities. These supplementary searches were intended to support interpretation and contextualization rather than to create an exhaustive systematic review evidence set.

IEEE – Institute of Electrical and Electronics Engineers; MeSH – Medical Subject Headings; MEDLINE – Medical Literature Analysis and Retrieval System Online; TS – Topic Search.
